# Bioinspired Hierarchical Architecture with Water Transport Channels for Strong Adhesion at the Sweating Interface

**DOI:** 10.1002/advs.202524043

**Published:** 2026-04-07

**Authors:** Jieliang Zhao, Yu Xiang, Heng Wang, Huiqing Xu, Zhong Liu, Lulu Liang, Fengwen Jiang, Xuemei Chen, Wenzhong Wang, Chenqing Liu, Guodong Liu, Shaoze Yan, Xu Hou

**Affiliations:** ^1^ School of Mechanical Engineering Beijing Institute of Technology Beijing P. R. China; ^2^ Institute of Advanced Technology Beijing Institute of Technology Jinan P. R. China; ^3^ College of Otolaryngology Head and Neck Surgery Chinese PLA General Hospital Beijing P. R. China; ^4^ Department of Mechanical Engineering Tsinghua University Beijing P. R. China; ^5^ Institute of Electrochemical Science and Engineering State Key Laboratory of Physical Chemistry of Solid Surfaces College of Chemistry and Chemical Engineering Xiamen University Xiamen P. R. China

**Keywords:** bio‐inspired interface, high permeability, liquid transport, wet adhesion

## Abstract

The persistent conflict between solid adhesion and liquid management fundamentally limits the reliability of epidermal electronics, as sweat accumulation disrupts interfacial integrity and sensor signal fidelity. This study presents a paradigm‐shifting strategy that physically decouples these requirements: a composite membrane patch with bioinspired hierarchical hydration architecture for sweat‐immune sensing. Inspired by the adhesive systems of tree frogs and bees and the fluid‐handling capabilities of bird beaks and pitcher plants, the architecture employs self‐regulating triphase liquid bridges to enhance adhesion. Hierarchical tapered microgrooves generate substantial Laplace pressure (>800 Pa) for directional liquid transport (up to 500 mm/s). This synergistic action maintains a dynamically balanced hydration environment at the skin interface, achieving a water vapor transmission rate 2.7‐fold higher than commercial patches. Consequently, it enables continuous monitoring of electrophysiological signals and sweat biomarkers, even during profuse sweating. This design principle offers a generalizable platform for developing robust, high‐performance epidermal devices.

## Graphical abstract

To address adhesion failure in sweaty epidermal electronics, this study proposes a cross‐species, multi‐scale biomimetic interface. By modeling staged wet adhesion, it integrates self‐regulating liquid bridges with directional drainage. This design ensures stable, high‐performance flexible sensing in complex physiological environments, offering a universal platform for robust wearable monitoring.

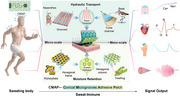

## 1 Introduction

Flexible sensors have revolutionized non‐invasive physiological monitoring by enabling continuous tracking of vital signs (e.g., electrocardiogram (ECG) [1], electromyogram (EMG) [2], electroencephalogram (EEG) [3], and galvanic skin response (GSR) [4]) and biomarkers [5, 6], advancing health management. Compared with rigid devices, flexible sensing systems offer superior comfort, skin conformability, and biocompatibility. However, maintaining long‐term reliability on sweat‐prone skin remains challenging. Sweat naturally secreted or retained at the device–skin interface can severely impair sensor performance by reducing interfacial adhesion, increasing electrical impedance and noise, degrading signal fidelity, and potentially promoting microbial growth that causes skin irritation [7, 8, 9]. These issues ultimately compromise the accuracy of health monitoring, the long‐term stability of the device, and user compliance. Therefore, it is essential to develop flexible sensing interfaces capable of directional sweat management. Such interfaces must maintain stable wet adhesion while actively managing moisture through efficient liquid–vapor exchange, thereby establishing a dynamically balanced microenvironment.

**FIGURE 1:**
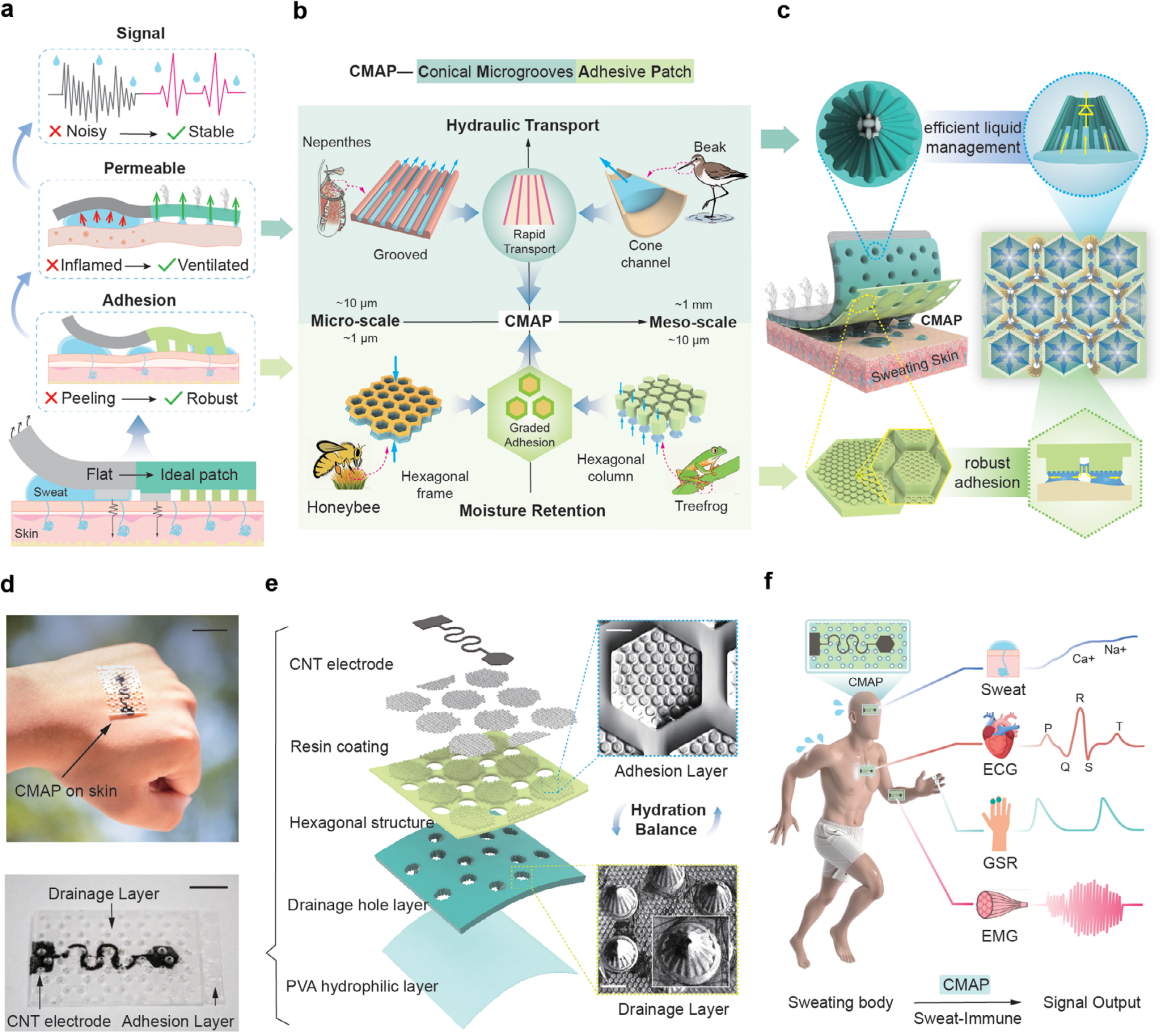
Bioinspired wearable Conical Microgrooves Adhesive Patch (CMAP): integrated signal acquisition, wet adhesion, and sweat management. a) Performance comparison between ideal patches and traditional patches on sweaty skin surfaces: Ideal patches exhibit high adhesion, breathability, and moisture absorption, significantly enhancing signal stability. b) Design inspiration of the CMAP: tapered capillary‐driven flow in waterfowl beaks and lubricated transport structures of pitcher plant peristomes (for drainage); composite hexagonal structures of bee/tree frog footpads (for wet adhesion). These elements work synergistically to achieve a balance between adhesion and sweat removal. c) A schematic diagram of CMAP application and its textured surface with adhesion and drainage zones. The drainage and adhesion structures are designed to enable efficient liquid management and strong adhesion capabilities for CMAP. d) Photograph of the CMAP worn on the human body (scale bar: 2 cm) and image of the physical device (scale bar: 10 mm). e) Layered structure of the CMAP illustrating the adhesion and drainage functional layers. The integrated array design of the bottom adhesive region (scale bar: 80 µm) and drainage micropores (scale bar: 1 mm). f) The CMAP enables physiological signal measurement while managing sweat‐induced interference.

To enhance device–skin adhesion, bioinspired adhesive substrates have been widely explored [10]. By mimicking biological systems such as octopus suckers [11], gecko setae [12], and insect mushroom‐shaped suction cups [13], researchers have successfully fabricated micro/nanostructured patches with enhanced adhesion performance. However, a critical design limitation of these bioinspired adhesive interfaces lies in their insufficient environmental permeability. These interfaces often act as barriers, trapping sweat at the interface. The resulting liquid accumulation can severely weaken adhesion strength, increase interfacial electrical impedance, introduce noise, and significantly degrade the quality of bioelectrical signals (Figure 1a). Therefore, achieving a synergy between stable adhesion and efficient liquid transport represents a central challenge in flexible interface engineering. Recent strategies have focused on increasing substrate porosity, improving liquid transport pathways, and developing novel liquid‐based gating technology [14, 15]. Compared with conventional films, approaches utilizing porous networks [16], nanofiber composites [17], and microchannel arrays have enhanced sweat clearance rates by several orders of magnitude [18, 19]. Although these advances alleviate practical transport limitations, a gap remains in the design of integrated coupling mechanisms.

Nature offers abundant design inspiration for multi‐mechanism‐driven directional liquid transport. Typical examples include the tapered beak of waterfowl that drives autonomous capillary flow, and the hierarchical microgrooves of the pitcher plant that enable ultraslippery transport (Figure 1b) [20, 21]. Though such principles inspire wet‐state pumping designs, achieving both stable wet adhesion and effective sweat removal remains difficult. To address this, we propose a composite bioinspired strategy that synergistically integrates the liquid‐bridge adhesion mechanism of tree frog/bee footpads, the tapered capillary transport principle of waterfowl beaks, and the lubricated low‐resistance transmission features of the pitcher plant peristome (Figure 1b), thereby overcoming the intrinsic trade‐off between adhesion and sweat transport. This strategy establishes a multiscale heterogeneous microsystem that enables efficient directional removal of sweat from the critical device–skin interface by regulating gradient capillary forces and wettability (Figure 1c). We designate this composite interface as the Conical Microgrooves Adhesive Patch (CMAP) (Figure 1b,c).

The core innovation of CMAP lies in integrating a hexagonally arranged micropillar array inspired by tree frog and bee footpads (Figure 1b), establishing a structural foundation for controllable wet adhesion [22, 23]. While maintaining strong adhesion under humid conditions, the design incorporates tapered geometries from waterfowl beaks and hierarchical grooves from the peristome of pitcher plants, forming a bioinspired microchannel system. This topological design physically decouples adhesion and drainage: micropillars ensure interfacial adhesion, while microchannels actively divert excess sweat, preventing failure from liquid bridge coalescence common in homogeneous structures (Figure S1). The coupling between the adhesive and drainage layers enables multifunctional synergy (Figure 1b). Integrated with biocompatible CNT electrodes, the patch enhances signal fidelity and enables simultaneous monitoring of sweat ions, ECG, GSR, and EMG on sweating skin (Figure 1f). This structure establishes a new paradigm of dynamic hydration balance—“moisture‐controlled adhesion, adhesion‐guided transport” (Figure S2)—offering a robust solution to sweat interference and a biomimetic foundation for next‐generation high‐performance interface platform.

Bioinspired Hierarchical Hydration Architecture offers a significant advancement in achieving sweat‐immunity for high‐performance epidermal sensing. Conventional bioadhesives suffer from a fundamental trade‐off, where breathability is sacrificed for adhesion, resulting in sweat accumulation and signal degradation. CMAP overcomes this by mimicking the adhesive specialization of tree frog and bee footpads and the fluidic guidance of bird beaks and pitcher plants. Its design autonomously regulates moisture: microscale liquid bridges secure robust adhesion, while gradient microgrooves propel sweat away from the interface with Laplace pressure‐driven flow (800 Pa, 500 mm/s). This ensures a stable skin interface with superior breathability (Water Vapor Transmission Rate (WVTR) 2.7x commercial ECG patches), permitting uninterrupted, high‐fidelity acquisition of physiological electrical signals and sweat ion dynamics. Our work establishes a scalable framework for engineering sweat‐resilient interfaces that are critical for continuous monitoring.

## 2 Results

### 2.1 Adhesion Enhancement through Microtexture Fragmentation

**FIGURE 2:**
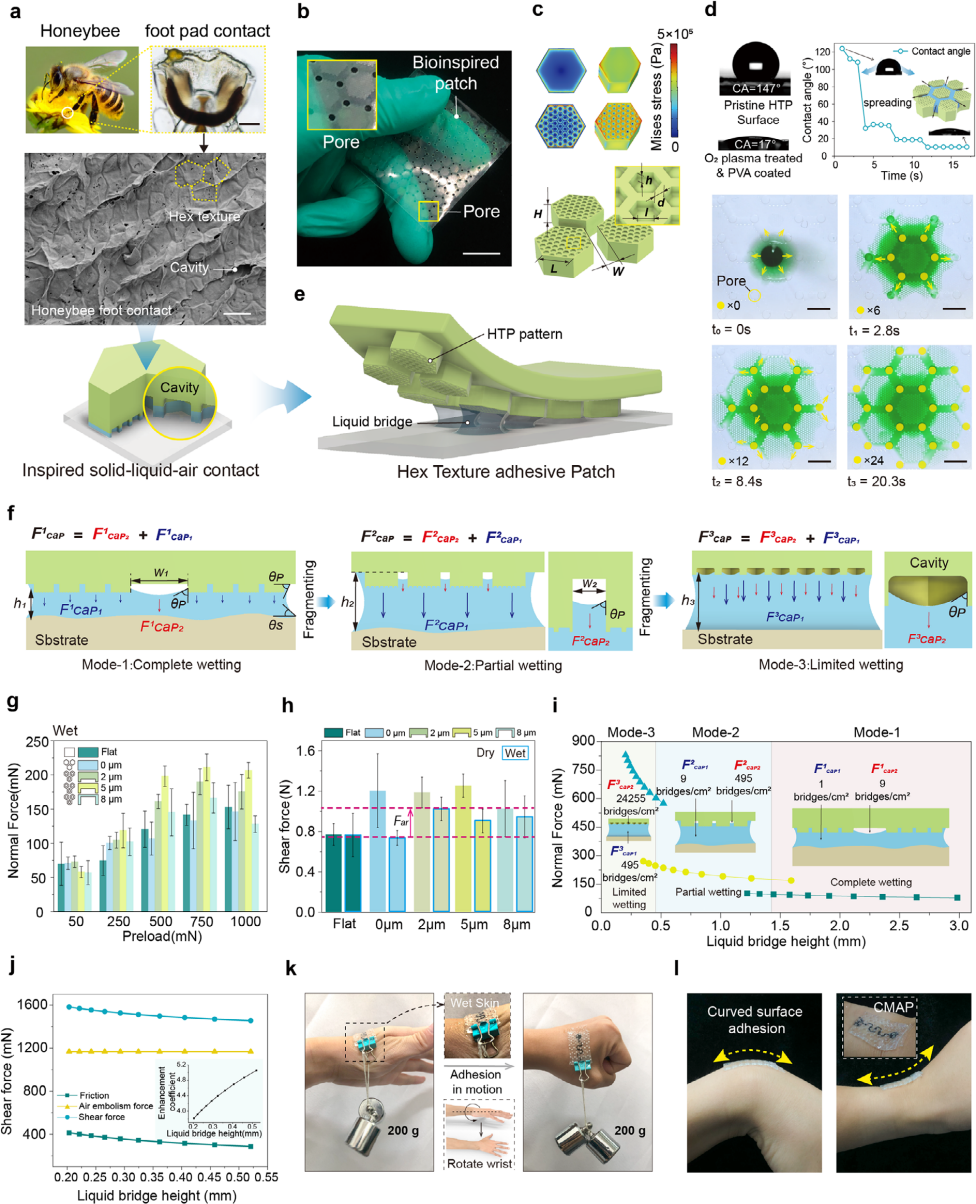
Bioinspired design, liquid regulation, and adhesion performance of the HTP. a) Morphological inspiration from bee footpad adhesion. Cryo‐SEM images of bee footprints inspired the design of the three‐phase contact interface. b) Optical image of the physical HTP patch showing the array of drainage pores on its surface. c) Finite element analysis (FEA) stress distribution of the HTP adhesive unit and corresponding dimensional parameters. d) Comparison of wettability (contact angle) and wetting spread between untreated and O_2_ plasma/PVA‐treated HTP surfaces. (scale bar: 2.5 mm). e) Schematic of liquid bridge formation between the bioinspired HTP and a planar substrate, inspired by the bee footpad. f) Schematic illustration of the three‐stage normal wet adhesion mechanism based on liquid bridge rupture in the HTP pattern. g) Relationship between preload and normal force on flat surfaces and surfaces with grooves of different depths. h) Comparison of shear force under dry and wet conditions for different surface textures. i) Model prediction of forces generated by the hierarchical adhesion mechanism and the number of curved liquid bridges per unit area at different bridge heights. j) Shear force–displacement curves regulated by the air‐plug effect, along with the corresponding variation in normal force enhancement factor. k) Demonstration photograph showing an enhanced CMAP suspending a 200 g weight on moist, moving skin. l) Demonstration of stable adhesion of the CMAP on curved skin surfaces.

Tree frogs and bees adhere to wet vertical surfaces via solid–liquid–gas three‐phase interfaces formed by micron‐scale pillars and concave hexagonal footpad textures, achieving shear adhesion in the kilopascal range (Figure 2a) [23, 24, 25]. Inspired by this, we developed a bioinspired composite pattern (adhesive layer component of CMAP)—HTP (Honeybee–Tree Frog Pattern, Figure 2e)—with a three‐level topological structure mimicking these features. Level 1: A macroscopic hexagonal framework combines solid adhesive zones with hollow drainage grooves and pore nodes at vertices, forming a vertical microchannel network for coordinated adhesion and sweat clearance (Figure 2b). Level 2: A micropillar array of sub‐millimeter hexagonally arranged pillars (density: 60%; diameter *L* = 120 µm; height *H* = 96 µm; spacing *W* = 60 µm; *H/L* = 0.8; *H/W* = 1.6; Figure 2c; Confocal laser scanning microscope (CLSM) data in Figure S3) generates capillary bridges providing adhesive force. Level 3: Nanocavities with concave hexagonal geometry (*l* = 12 µm) stabilize air bubbles and reinforce the three‐phase contact interface, enhancing adhesion. Finite element analysis shows this framework reduces edge stress (Figure 2c), while the nanocavities induce localized buckling, dissipating stress and lowering interfacial shear stress to *τ_max_* = 0.24 MPa, preventing delamination. Cyclic testing (1000 cycles) confirmed this robustness, showing intact pillars with only minor superficial wear rather than structural buckling (Figure S4).

The hexagonal texture optimizes in‐plane fluid transport performance, where drainage grooves at the vertices and vertical through‐holes together form a sweat gland–inspired multilevel transport network (Figure 2b). This design enhances the efficiency of directional sweat transport and significantly reduces the risk of sweat gland blockage. After surface modification with oxygen plasma–polyvinyl alcohol (O_2_ plasma–PVA), the contact angle decreased by 88% (Figure 2d). Notably, the contact angle increased by only ∼30% after 24‐h immersion and ∼20% after 500 bending cycles (Figure S5). The hydrophilic micropillar array and through‐holes synergistically drive directional sweat transport (Figure 2d), allowing sweat to rapidly redistribute along predefined pathways and evaporate uniformly through the porous structure, effectively preventing local liquid accumulation. This design enables an in‐plane liquid transport rate of up to 4.03 µL·cm^−^^2^·s^−^^1^ on the HTP surface, effectively avoiding localized fluid buildup and ensuring reliable adhesion under humid conditions.

To investigate the wet adhesion enhancement of the HTP's multiscale structure, we used fluorescence microscopy to visualize dynamic liquid film rupture (Figure S6) and developed a coupled structure–liquid–substrate model to quantify normal adhesion during peeling (Figure 2f). Adhesion is mainly driven by capillary force (*F_cap_*), which scales inversely with bridge height (*h*) (*F_cap_* ∝ 1/*h*) and with the fourth power of the three‐phase contact line length (*F_cap_* ∝ *l^4^*). The hierarchical structure regulates liquid morphology through a three‐stage rupture process: Stage I—Bridge formation: Liquid rises from grooves to pillar tops, forming microscale bridges and menisci (Figure S6‐i). Stage II— Bridge rupture and the three‐phase contact line (TCL) elongation: As the film ruptures, sub‐millimeter liquid bridges form. Though the contact area decreases by 15.6%, bridge number increases 54‐fold, and total TCL length grows by 214%. Stage III—Air plug stabilization: Drainage and evaporation form bubbles within the bridges, creating a stable air‐plug interface that extends and reinforces the TCL (Figure S6‐v).

The three enhancement mechanisms—bridge multiplication, TCL elongation, and stabilized air‐plug formation—are quantitatively captured by a normal force model derived from a modified Young–Laplace equation [26, 27]:

(1) $$f_{cap}=f_{1}+f_{2}=A\cdot \frac{\gamma \left(\cos\theta_{t}+\cos\theta_{b}\right)}{h}+\gamma l$$

here, *f_1_* and *f_2_* represent capillary forces from the meniscus and three‐phase line tension, respectively. *A* is the wetted area, *γ* is the liquid surface tension, *θ* the contact angle, *h* the bridge height, and *l* the wetted perimeter. Across the three rupture stages, changes in *A*, *h*, and *l* modulate the wet adhesion force dynamically.

To clarify the HTP's wet adhesion mechanism, we developed a mechanical model describing its hierarchical structure interacting with the liquid–glass substrate (derivations in Note S2). The model identifies two capillary components: the liquid bridge force at the texture–substrate interface *F_cap1_* and the wetting‐induced force in the groove air gaps *F_cap2_*, both arising from meniscus tension and solid–liquid–gas contact lines. The total normal adhesion force is thus expressed as [27, 28]:

(2) $$F_{n}=F_{cap}^{1}+F_{cap}^{2}$$

(3) $$F_{cap}^{1}=n_{1}\;\left[\frac{\gamma \cdot \left(cos\theta_{p}+cos\theta_{b}\right)}{h}\cdot A_{h}+l_{h}\cdot \gamma cos\theta_{p}\right]$$

(4) $$F_{cap}^{2}=n_{2}\;\left(\frac{2\gamma \cdot cos\theta_{p}}{W}\cdot A_{w}+l_{w}\cdot \gamma cos\theta_{p}\right)$$

In this model, *n* is the number of structural units and *A* the wetted area per liquid bridge. As evaporation and suction drive fluid migration from secondary grooves (Figure S7), both the number and geometry of bridges change. Based on volume conservation, the HTP exhibits three wetting modes on a 15 mm^2^ patch: fully wetted (24 µL), partially wetted (0.8 µL), and minimally wetted (0.023 µL) (Figure 2i). Model predictions and critical bridge height estimates (Note S1) show that in the minimal mode (*h* < 0.25 mm), adhesion peaks at 850 mN. In all modes, adhesion decays exponentially with increasing liquid volume, stabilizing thereafter. The decay accelerates with wetting level, with exponents *β_1_* = 2.4 (fully), *β_2_ =* 13.4 (partially), and *β_3_* = 16.0 (minimally wetted).

The relative contributions of the capillary force components (*F*_cap1_ and *F*_cap2_) dynamically vary with the height of the liquid film *h* and its degree of rupture. When the contact angles of the hierarchical structure and the substrate are comparable (*θ_texture_ ≈ θ_substrate_*), the capillary force ratio (*η_cap_*) can be simplified as:

(5) $$R\;t_{cap1}=\frac{F_{cap}^{1}}{F_{cap}^{1}+F_{cap}^{2}}={\left[1+\frac{n_{i+1}\left(\frac{A_{w}}{W}+l_{w}\right)}{n_{i}\left(\frac{A_{h}}{h_{crit}}+l_{h}\right)}\right]}^{-1}$$

The capillary force is governed by a characteristic dimension *κ*, defined as:$\kappa =n(\frac{A}{d}+l)$. This parameter determines the relative contributions of *F_cap1_* and *F_cap2_* (Figure S8). Under fully wetted conditions, both forces contribute equally (∼50%). As liquid volume decreases, bridges shift from planar to discrete contact (Figure S6‐iii), increasing total adhesion. In the minimally wetted state, *F_cap2_* dominates (>70%), as the groove network enhances three‐phase contact line density. Thus, macroscopic adhesion arises from the synergy of *F_cap1_* and *F_cap2_*, while microscale enhancement stems from liquid self‐regulation and contact line densification by the groove structure.

When the liquid film height drops below 500 µm, the HTP undergoes tertiary rupture, forming discrete air cavities between micropillars (Figures S6‐v and S7a). This resembles the film regulation in bee footpads, where geometric depressions guide liquid redistribution to enhance capillarity. Under shear, asymmetric meniscus curvature arises across the cavities, triggering the air‐plug effect. This stems from shear‐induced contact angle hysteresis (advancing *θ_a_* ≈ 5°, receding *θ_r_* ≈ 20°; Figure S7b), producing an asymmetric Laplace pressure. The resulting pressure gradient generates a “piston thrust” driven by surface energy difference and can be expressed as:

(6) $$\begin{array}{c} \delta =\Delta P_{right}-\Delta \;P_{left}=\frac{\gamma }{h}\;\left[\left(\cos\theta_{ft}+\cos\theta_{fb}\right)-\left(\cos\theta_{rt}+\cos\theta_{rb}\right)\right] \end{array}$$

Based on the topology of the gas cavity network, the number of gas cavities per unit pillar in orthogonal directions is defined as *n_1_* and *n_2_*, with their effective totals approximated as $N_{1}\approx \sqrt{n_{1}},N_{2}\approx \sqrt{n_{2}}$. Let *S_δ_* denote the axial cross‐sectional area of a single gas cavity; then the embolic force can be expressed as:

(7) $$F_{af}=N_{1}\;N_{2}\sum_{n=1}^{N_{i}N_{2}}n\cdot \delta \cdot \;S_{\delta }=\frac{N_{1}^{2}N_{2}^{2}\left(N_{1}N_{2}+1\right)}{2}\;\cdot \delta \cdot S_{\delta }$$

HTP's tangential adhesion combines normal friction and retention from the air‐plug effect. This cavity‐induced enhancement is independent of liquid bridge height, providing strong directional robustness under dynamic wet conditions. To quantify this effect, we define a normal force enhancement factor as:

(8) $$e=\frac{\mu \cdot F_{n}+F_{af}}{\mu \cdot F_{n}}=1+\frac{F_{af}}{\mu F_{n}}$$

here, *µ* is the friction coefficient between structure and substrate. The air‐plug effect enhances HTP shear resistance 4–5 times Figure 2j). Adhesion was characterized via dynamic loading (Figure S9). Under controlled wetting (5 µL·cm^−^^2^) and varying preload, normal adhesion increased with load, saturating at ∼500 mN (Figure 2g). Samples with concave cavities (depth ≥ 2 µm) showed superior adhesion, confirming the reinforcement role of microcavities. Tests at different wetting levels (0, 5, 10 µL·cm^−^^2^; Figure S10) revealed that cavity‐containing samples maintained greater adhesion at high humidity. The concave cavities optimize liquid bridge formation and stabilize the TCL, preventing lubrication failure under excess liquid.

Shear tests revealed distinct wetting responses of the microstructures (Figure 2h). Under dry conditions, cavity‐free hexagonal pillars exhibited higher shear strength due to larger solid contact and efficient stress distribution. In contrast, under humid conditions (10 µL·cm^−^^2^), cavity‐containing structures enhanced tangential force via air trapping, consistent with the described mechanism. The three‐level modulation of liquid bridge height highlights the role of hierarchical micro/nanostructures and guides biomimetic design: matching structural liquid retention with target bridge heights optimizes adhesion. Low‐adhesion Modes I–II suit rapid detachment (e.g., reusable tapes), whereas Mode III supports high load‐bearing applications (e.g., wet‐surface grippers). Figures S11 and S12 demonstrate HTP's adhesion robustness across various substrates under low humidity.

A biocompatible resin layer was applied to the HTP to meet skin adhesion requirements. Its micro/nanotexture enables drainage and adaptive adhesion, allowing the patch to support a 200 g weight on moist skin while maintaining stability during rotation (Figure 2k). This load‐bearing capability is mechanically sustained for extended periods provided that the interfacial peeling angle remains below the critical detachment threshold. Furthermore, the patch conforms to curved areas such as the wrist (Figure 2l). To substantiate this adaptability, quantitative porcine skin tests mimicking the finger (*R* = 10 mm), thigh (*R* = 65 mm), and shoulder (*R* = 180 mm) confirmed robust adhesion, with normal forces of ∼3.8–4.3 N and peel resistance of ∼0.3–0.5 N (Figure S13). These capabilities provide a functional basis for reliable adhesion and physiological signal acquisition under load, moisture, and curvature.

### 2.2 Enhanced Unidirectional Diffusion in Microgrooved Conical Channels

Human sweat, essential for thermoregulation, can reach 2.3 L/day [29]. Sweat accumulation at the patch–skin interface causes contact failure, increases resistance, lowers signal‐to‐noise ratio, and accelerates electrode corrosion, exposing the limitations of conventional patches in liquid management. Inspired by directional transport in pitcher plants, cacti, and desert beetles [21, 30, 31, 32, 33], asymmetric tapered structures improve permeability via channel pressure differences [3, 34], but face two main drawbacks: transport rates <200 µm/s and poor durability of hydrophilic coatings (O_2_ plasma‐treated, contact angle >100° after two days) [1], limiting long‐term use in flexible devices.

We propose a synergistic design combining a gradient‐wettability surface with a bioinspired microchannel network to achieve directional sweat transport via capillary forces while maintaining interfacial hydration balance through optimized fractal pathways. Numerical analysis of sweat filling in tapered pores is in Note S3. Theoretical analysis shows transport dynamics depend on cone angle *α*, contact angle *θ*, and inlet radius *R_1_*, with capillary force expressed as follows (Note S4 and Figure S11):

(9) $$F_{L}=\Delta P\cdot S=\frac{2\gamma \cdot \cos\left(\theta -\alpha \right)}{R_{1}}\;\cdot \pi \;R_{1}^{2}=2\pi R_{1}\gamma \cdot \cos\left(\theta -\alpha \right)$$

**FIGURE 3:**
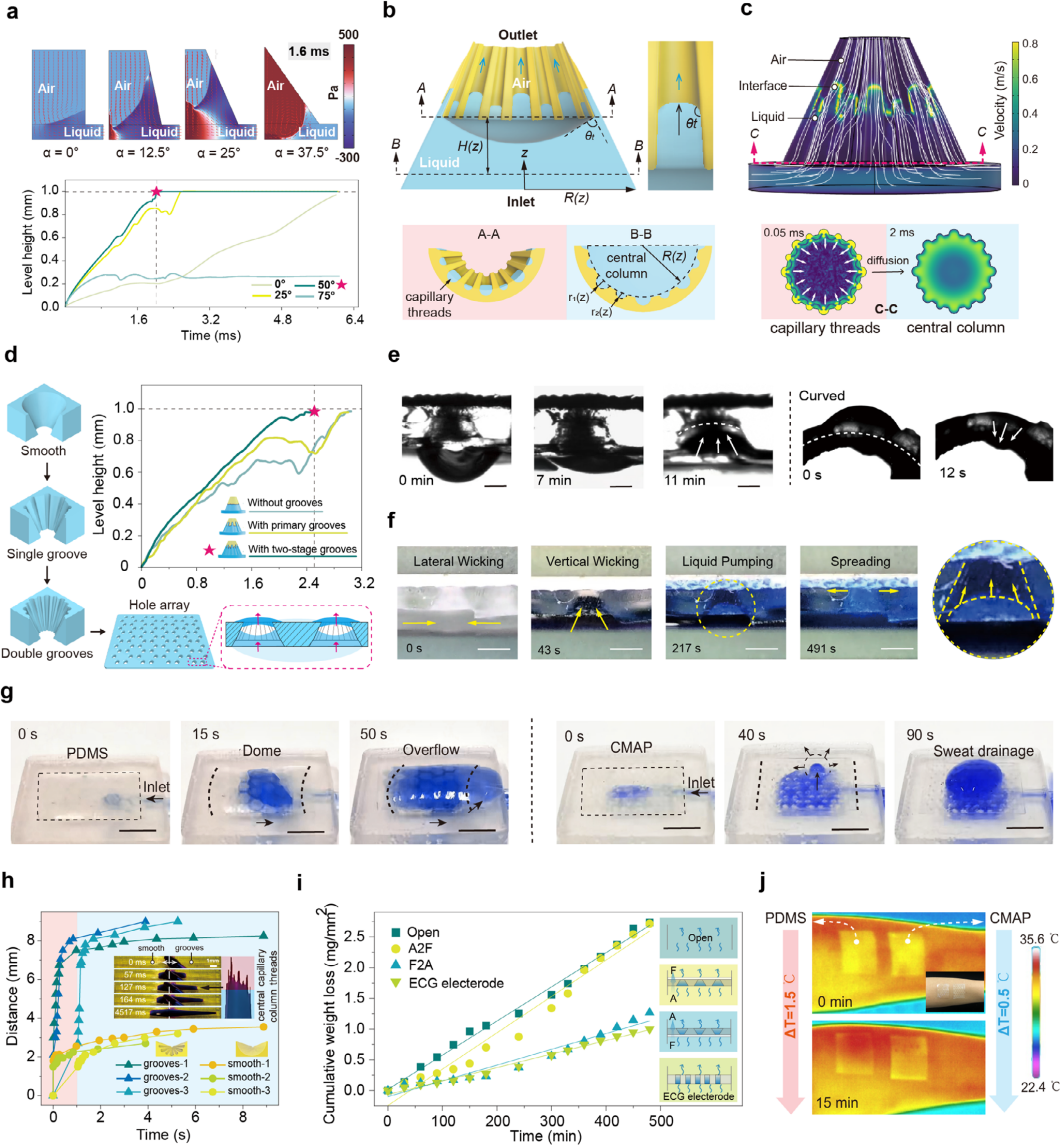
Water transport mechanisms and permeability analysis in microgroove tapered channels. a) Pressure distribution and liquid height variation during a 1.6 ms water transport process for channels with identical inlet radii but half‐cone angles of 0°, 12.5°, 25°, and 37.5°. b) Schematic of water transport along the microgroove channel, with the central liquid surface depicted as a curved meniscus. Upward movement occurs in two stages: Stage I—filling along the microgroove; Stage II—rise of the main liquid meniscus. c) Simulated streamlines and velocity distribution during liquid rise in the microgroove channel. At the inlet cross‐section, high‐speed liquid rises along the surrounding microgrooves, gradually driving the central liquid meniscus upward. d) Transport capacity differences generated successively by smooth holes, single‐groove holes, and double‐groove holes; the arrayed perforated membrane enables transmembrane water transport. e) Water transport process of CMAP when placed horizontally against gravity and under bending conditions. (scale bar: 0.5 mm) f) Process of the liquid film transporting from the bottom through the CMAP to the top fabric layer. (scale bar: 1 mm) g) Comparison of CMAP and PDMS adhesion on a simulated sweat‐secreting surface. (scale bar: 15 mm) h) Horizontal water transport influenced by the microgroove structure, exhibiting unidirectional transport behavior while propagating at a faster speed. i) Water evaporation loss over time; linear regression indicates the water vapor transmission rate. j) Comparison of maximum temperature change on the skin after 15 min of attachment between unpatterned PDMS patches and CMAP patches.

Finite element simulations (Figure 3a and Figure S15) show that a capillary channel with optimized geometry (half‐cone angle *α* = 25°, height 1 mm, tapering diameter 1.6–0.5 mm, *Δd*  = 1.1 mm/mm) generates a Laplace pressure up to 800 Pa, driving an average sweat transport rate of 500 mm/s—≈3 times faster than a vertical capillary. During filling, capillary force (*F_cap_* ≈ 6.2 mN) far exceeds gravitational force (*F_grav_* ≈ 5.3 × 10^−^^3^ mN), effectively preventing backflow. The simulation was conducted under the specific constraints and loading conditions summarized in Table S1.

Pitcher plant–inspired multi‐scale microgrooves were added to channel walls (Figure 3b) [35, 36, 37, 38], with primary/secondary radii 0.09/0.06 mm, spacing 0.25 mm, eight‐fold circumferential periodicity, and axial radius taper *ΔR* = 0.03 mm/mm. This curvature‐gradient induces capillary pressure, forming a precursor liquid film that enhances spreading and shifts transport from full liquid–solid to partial liquid–liquid contact. Simulations (Figure 3c and Figure S18) show two‐stage dynamics: Stage I (0–0.1 ms)—the film spreads axially within microgrooves (coverage 58.8%), shortening solid–liquid contact and reducing friction; Stage II (>0.1 ms)—under 500 Pa capillary pressure, the lubricating film induces super‐slippery behavior, reducing contact angle from 65° to ∼0° and achieving 389 mm/s, a 12% improvement over smooth surfaces (Figure 3d; Figures S16 and S17). For HTP integration, microgroove pores were arranged in a hexagonal array to form a water transport membrane.

**FIGURE 4:**
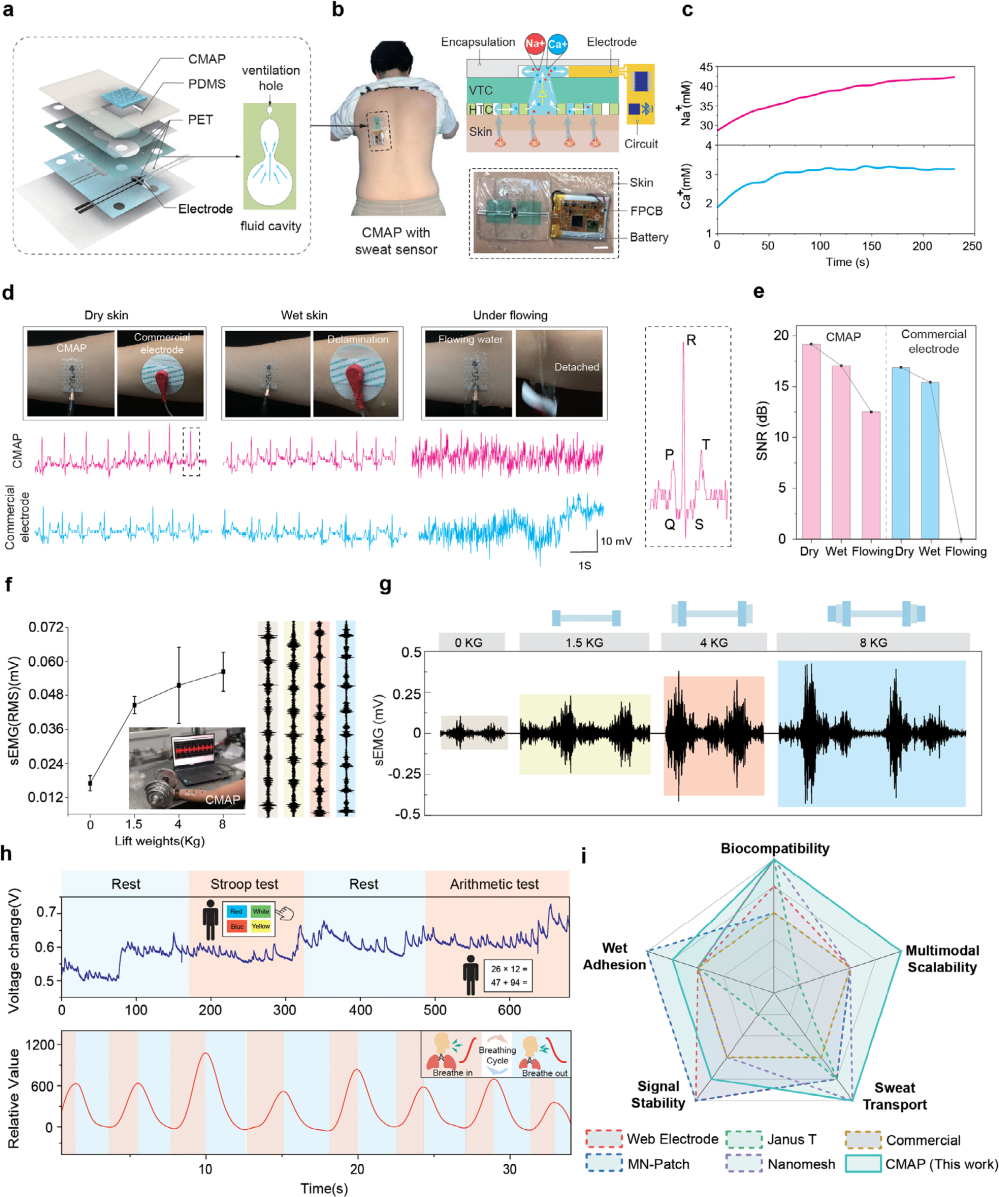
Applications of CMAP in sweat analysis and physiological signal monitoring. a) Exploded schematic of the CMAP integrated with a sweat‐sensing module. b) Shows a photograph of a CMAP patch with an integrated sweat‐sensing circuit performing real‐time sweat monitoring on human skin, along with its working mechanism. Here, VTC stands for Vertical Transport Channel and HTC represents Horizontal Transport Channel. c) Temporal profiles of Ca^2^^+^ and Na^+^ ion concentrations in the collected sweat. d) Comparison of signal acquisition performance between CMAP and commercial patches under various wet conditions, along with representative ECG waveforms. e) Comparison of ECG signal‐to‐noise ratio (SNR) measured by CMAP electrodes and commercial electrodes on skin under different moisture levels. f) EMG root‐mean‐square (RMS) values and corresponding signals recorded by CMAP during bicep curls with different weights. g) Correlation between specific load weights and signal amplitudes. h) Demonstration of CMAP applied for monitoring human respiration amplitude and recording galvanic skin response (GSR) signals. i) Comparison of adhesion, biocompatibility, sweat transport capability, and sensing performance among CMAP, Web Electrode [3], MN‐Patch [34], Janus‐T [18], Nanomesh [9], and commercial electrodes.

To test unidirectional, anti‐gravity transport, 10 µL droplets were applied to the large‐pore side of the composite membrane (Figure 4e). In both horizontal and downward‐bent configurations, droplets spontaneously moved to the small‐pore side. Horizontally, transport completed in 11 min; downward‐bent, it took 12 s due to gravity and passive pore expansion. These results confirm robust water transport under curved and vertical conditions. In arrayed membrane tests (Figure S22), the CMAP guided liquid horizontally into tapered pores, then upward via capillarity (Figure 3f), demonstrating cooperative transport. CMAP also shows superior gas and sweat permeability compared to PDMS, with drainage structures distributing sweat horizontally and extruding it through tapered pores, preventing interface bulging or detachment under perspiration (Figure 3g and Figure S23).

Horizontal transport tests show that layered microgroove channels achieve pronounced anisotropic transport via multistage capillary films (Figure 3h). Joined with a smooth α = 15° channel, 2.5 µL droplets preferentially propagated along the microgroove side (0–1 s, v = 420 mm/s). At 127 ms, wetting reached 8.1 mm vs. 3.3 mm on the smooth side (∼2.7:1); by 4.5 s, the microgroove side was fully wetted, smooth side 43%. Anisotropy factor ξ = 0.57 (ξ  = (*d*_+_ − *d*_−_ )/*max*(*d*_+_,*d*_−_)) (pitcher‐plant ξ = 0.67) confirms strong directional transport [33]. Two‐stage spreading occurs: Stage I (0–1 s), capillary pumping peak 500 mm/s; Stage II (>5 s), after depletion, the microgroove effect diminishes and transport approaches smooth‐channel behavior. Vertical transport (Figure S20) shows multistage microgrooves reduce transport time by 67% (smooth 3 s, microgroove 1 s).

HTP film WVTR was measured under heated conditions per modified ASTM E96‐95 and compared with a commercial ECG patch (XUNDA X‐1) [3]. Linear regression (R^2^ > 0.95) shows the adhesive–fiber (A2F) layer has WVTR (5.91 ± 0.28) × 10^−^^6^ mg·mm^−^^2^·min^−^^1^, close to the open‐system value (5.55 ± 0.13) × 10^−^^6^ mg·mm^−^^2^·min^−^^1^ (Figure 3i). Evaporation occurs in two stages: 0–2 h—low rate due to droplet accumulation; 2–6 h—increased rate via capillary pumping from condensation. The A2F layer's WVTR is 2.7‐fold higher than the commercial patch, confirming superior moisture permeability. Ventilation‐hole arrays also enhance thermal comfort, with central temperature rising only 0.5°C after 15 min of attachment (Figure 3j), also lower than a simple perforated control (Figure S19).

The surface microstructure controls evaporation via contact line pinning (Figure S21). Microgrooves stabilize the pinned three‐phase line, maintaining a constant wetted area while contact angle decreases from 90° to 0°, prolonging liquid film lifetime. In contrast, the control lacks pinning, causing rapid contact‐area shrinkage (volume‐to‐area ratio 0.027 → 0.068 mm^−^^1^) [39]. Hydrostatic tests show pronounced unidirectional permeability^3^ (Figure S25): critical penetration pressure in the fiber‐to‐adhesive (F2A) direction is 17.0 mm H_2_O (167 Pa), 3.1‐fold higher than adhesive‐to‐fiber (A2F, 5.5 mm H_2_O, 54 Pa). Notably, A2F penetration is 80% lower than commercial patches, offering a solution for highly breathable wearables.

### 2.3 Physiological Signal Acquisition of CMAP

CMAP was integrated into a sweat‐sensing patch comprising a PDMS substrate and a 0.2 mm PET microchannel layer [40]. Its backside features microchannels and venting pores (∼500 µm, Figure 4a) that guide sweat to the sensing region. Human back‐wear tests (Figure 4b) showed the sensor/circuit system reached signal saturation within ∼240 s, measuring Na^+^ (43 ± 0.5 mm) and Ca^2^^+^ (3.2 ± 0.1 mm) in sweat (Figure 4c). Calibration was performed with standard ion solutions (Figure S26). To validate performance in complex sweat matrices, interference analysis confirmed high selectivity against competing ions (log *K_Na,K_* = −3.441, log *K_Ca,K_* = −2.585) and stable sensitivity across the physiological pH range of 4–8 (*R^2^* > 0.985) (Tables S2 and S3).

The CMAP‐integrated device also records bioelectrical signals. Interface impedance characterization (1 Hz–1 kHz) first confirmed that CMAP maintains stable electrical contact under both dry and wet conditions (Figure S27). With this stable interface, the device captures real‐time ECG using standard lead I electrodes (RA/LA/RL). Under simulated wet conditions (1 mL/cm^2^) with water flow (∼25 mL/s), CMAP electrodes maintained high‐fidelity PQRST waveforms and SNR ≈ 15–19 dB (Figure 4d,e) [41], outperforming commercial wet electrodes (e.g., XUNDA X‐1), which showed signal degradation and adhesion failure. This robustness extends to dynamic physical activities; during wrist flexion and body movements, CMAP effectively suppressed motion artifacts and baseline drift that severely distorted signals in commercial counterparts (Figure S28).

CMAP was attached to the arm to record sEMG during bicep curls with varying weights (0, 1.5, 4, 8 kg; Figure 4f). The flexibility and adhesion of CMAP allowed conformity to skin deformation. sEMG amplitude increased with load, showing a clear linear correlation between weight and RMS values, with distinguishable waveforms for different loads (Figure 4f,g).

CMAP also stably monitored respiratory amplitude (Figure 4h), with rising and falling phases corresponding to inhalation and exhalation exhibiting a consistent periodic pattern, reflecting reliable interfacial stability. Furthermore, for electrodermal activity (EDA) testing, CMAP was placed on the index and middle fingers to monitor mental stress during Stroop and countdown‐based arithmetic tasks. These activities elicited observable EDA responses (Figure 4h); although detailed analysis requires further study, these results demonstrate CMAP's potential for psychophysiological state recognition via GSR signals. Furthermore, a 24‐h wear test confirmed superior biocompatibility, showing a healthy skin interface free from the maceration and erythema observed with commercial electrodes (Figure S29). Figure 4i and Table S4 compare the adhesion, biocompatibility, sweat transport capability, and sensing performance of CMAP with other representative epidermal interfaces (Web Electrode [3], MN‐Patch [34], Janus‐T [18], Nanomesh [9]) and commercial electrodes, demonstrating that our CMAP outperforms in these key metrics. Detailed definitions of the scoring criteria for each performance indicator are provided in Table S5.

## 3 Conclusions

This study addresses the critical challenge of concurrent adhesion loss and signal degradation in flexible wearable sensors under moist conditions. By integrating wet adhesion mechanisms inspired by tree frog and bee footpads with directional liquid transport principles from avian beaks and pitcher plants, we developed a multiscale bioinspired composite interface. This interface sustains robust wet adhesion while efficiently removing sweat from the skin–sensor interface, establishing a dynamic hydration balance defined as “moisture‐regulated adhesion and adhesion‐guided drainage”.

Unlike prior strategies that optimized a single function, our approach physically partitions the topological structure to decouple and synergistically enhance adhesion and transport. We identified and leveraged the dynamic rupture mechanism of interfacial liquid films induced by hierarchical microstructures, maximizing the three‐phase contact line and triggering air‐cavity embolism effects, which significantly boost normal and shear adhesion. Simultaneously, the integrated bioinspired microchannel network uses capillary pressure from geometric gradients and a spontaneously formed lubricating precursor film to achieve rapid, low‐resistance unidirectional sweat transport, exceeding the efficiency of existing designs. This multi‐mechanism coupling offers a universal solution to liquid management challenges in flexible interfaces.

The practical utility of this interface was validated in multimodal physiological monitoring. Integrated sensor patches accurately and in real time measured sodium and calcium ion concentrations in human sweat and captured high‐fidelity ECG signals under simulated high‐humidity conditions, outperforming commercial electrodes. These results demonstrate effective mitigation of sweat‐induced interference, enabling uninterrupted, reliable health monitoring during physical activity and under elevated temperatures.

Sweat‐induced signal degradation remains a major obstacle for epidermal sensing. We resolve this with a CMAP that ensures signal stability through autonomous, directional sweat transport. Inspired by natural adhesive and fluidic systems, CMAP's hierarchical structure generates 800 Pa of laplace pressure, removing sweat at 500 mm/s while enhancing dry adhesion via liquid bridges. This dual mechanism maintains optimal skin‐device contact, outperforms commercial patches in breathability by 2.7‐fold, and enables sweat‐immune monitoring of electrophysiological and electrochemical signals. This strategy provides a generalizable blueprint for next‐generation high‐performance wearable electronics.

## 4 Methods

All experiments involving human subjects were reviewed and approved by the Ethics Committee of Beijing Institute of Technology. Informed written consent was obtained from all participants prior to the experiments.

### 4.1 Fabrication of CMAP

The PDMS prepolymer (Sylgard 184, Dow Corning) was mixed with the curing agent at a mass ratio of 10:1, degassed under vacuum for 30 min, and poured onto an SU‐8 mold. The mixture was spin‐coated (KW‐4BC, Beijing Cedes Electronics) with a high acceleration rate of 5000 rpm/s to ensure film uniformity, where the thickness was controlled within the range of 0.3–1.1 mm based on the specific spin speed (Figure S31), followed by curing at 75°C for 2 h [42]. The resulting PDMS bioinspired textured membrane was obtained by peeling off the mold. The conical‐pore membrane was fabricated via a gradient curing process: the PDMS prepolymer was injected into a conical‐pore mold, pre‐cured at 40°C for 30 min to retain structural integrity, and then fully cured at 80°C for 2 h [43]. After demolding, both the bioinspired textured membrane and the conical‐pore membrane were treated with oxygen plasma (50 W, 90 s). Finally, the bioinspired textured membrane was bonded to the plasma‐treated conical‐pore membrane under a pressure of approximately 0.1 MPa at 25°C for 30 min, achieving molecular‐level interfacial bonding via the self‐healing effect of PDMS (Figures S30, S33–S35). The robustness of this interface was quantitatively validated via destructive peel tests, where the observation of cohesive failure confirmed that the bonding strength exceeds the tensile strength of the PDMS matrix (Figure S32). Representative SEM images of the fabricated CMAP structure are presented in Figure S34.

### 4.2 Membrane Hydrophilic Modification

To achieve robust wet‐state adhesion and efficient sweat management, the PDMS surface was hydrophilically modified via oxygen plasma treatment combined with a polyvinyl alcohol (PVA) coating. First, oxygen plasma treatment (VP‐R3) was performed using optimized parameters (RF power: 160 W, oxygen flow rate: 250 mL/min, pressure: 0.1 MPa, duration: 90 s), reducing the water contact angle of the surface to below 20°. This treatment effectively eliminated residual viscoelasticity and introduced hydroxyl groups (–OH), significantly enhancing wettability to promote the formation of stable liquid bridges. Subsequently, a 1% wt. PVA coating was deposited by immersion for 10 min, followed by air drying [44].

### 4.3 Testing of Normal and Tangential Adhesion

Adhesion performance was evaluated using a portable tensile tester equipped with a micro‐force sensor (GSO‐500, Transducer Techniques) [45]. Samples (10 mm × 10 mm) were pretreated and then equilibrated in a humidity‐controlled chamber for 24 h. To ensure consistent adhesion conditions, the surfaces of both human and porcine skin were cleaned with 75% ethanol wipes and allowed to air‐dry completely prior to device attachment, thereby removing excess sebum and debris. Normal adhesion tests followed a preload–peel protocol: the patch was brought into contact with a glass substrate under a constant preload force (50–1000 mN) for 10 s, then separated vertically at a rate of ∼0.5 mm/s. Force–time curves were recorded, and peak adhesion forces were extracted. A humid environment was created by applying deionized water droplets onto the substrate. Shear adhesion tests were conducted by horizontally pulling the patch at a rate of ∼1 mm/s to determine the failure thresholds under both dry and wet (10 µL cm^−^^2^) substrate conditions. All tests were repeated five times to ensure the quantitative reliability of the bioinspired structure's adhesion performance under dry and wet interfacial conditions.

### 4.4 Water Vapor Transmission Rate Test

The sealing film was combined with different patch samples to seal serum bottles containing 12 mL of deionized water. All samples were simultaneously placed on a 50°C heating platform, with ambient temperature and humidity maintained constant at 26°C and 45%, respectively. The total mass of all samples was measured gravimetrically at approximately 25‐min intervals, and the water vapor evaporation permeability was derived by dividing the corresponding evaporated mass by the exposed area of the film.

### 4.5 Water Pressure Resistance Experiments

The membrane was tested using a two‐tube compression clamp. Dyed deionized water was gradually dripped into the setup, and the process was recorded on video for observation. When the dyed liquid began to seep downward, the height of the liquid column was recorded. The pressure at the bottom of the liquid column was then calculated and compared.

### 4.6 Sweat Monitoring Experiments

The integration of CMAP with a wearable electrochemical sensor first involves concentration calibration using standard‐concentration sweat. Subjects were made to perform exercise on an elliptical bicycle to induce sweat secretion, and a patch was applied to collect sweat stimulation. As sweat gradually fills the device over time, it enters the detection chamber via CMAP and interacts with the sensor, enabling observation of subsequent changes in the concentrations of Na^+^ and Ca^2^^+^ ions.

### 4.7 ECG and Respiratory Amplitude Test

In this study, lead I electrocardiography was used, with electrodes connected to the left arm (LA, left hand) and right arm (RA, right hand), and the left leg grounded, to reflect the potential difference of cardiac electrical activity between these two points. Physiological electrical signals were collected using a biosignal acquisition device (LabAide IX‐BIO4), which is equipped with four channels and automatically performs signal filtering and amplification after acquisition, enabling the generation of stable physiological signals from raw data. Lead I was used for ECG measurements, and the impact of patch performance on signal acquisition quality was evaluated by replacing the patch at the LA site and comparing results under different skin moisture conditions.

### 4.8 sEMG Measurements

sEMG signals were acquired using a custom‐built STM circuit board‐based device, with three electrodes placed adjacent to each other on the ventral side of the forearm for measurement. Subjects performed dumbbell curls in a seated position using weights of 0, 1.5, 4, and 8 kg. Each repetition consisted of approximately 5 s for complete lifting and lowering phases, followed by 5 s of relaxation. The curling exercise was repeated 5 times for each weight level, with a 5‐min rest period before proceeding to the next weight condition. The raw signals were filtered with a 50 Hz notch filter to obtain the final data.

### 4.9 GSR Measurements

Localized skin sweat loss was measured by attaching electrodes to the finger. Subjects were instructed to place their fingers on the electrodes and remain at rest for 3 min. They then performed a Stroop test to elicit mental stress, followed by another 3‐min rest period. Subsequently, a time‐limited two‐digit arithmetic test was administered. Throughout the procedure, the subjects maintained finger contact with the electrodes to enable continuous acquisition of electrical signals between the skin and the electrodes. Emotional variations were assessed based on sweat‐induced changes in the electrical signals.

## Funding

This work was supported by the National Natural Science Foundation of China (grant 52375282), the National Science and Technology Major Project of China (SKS‐2022031), Beijing Nova Program (20230484377, 20240484712), the BIT Teli Young Fellow Recruitment Program (RCPT‐20220005), HaiYou Industrial Experts Programme (CYLj20241901469), Shandong Provincial Natural Science Foundation (ZR2025QB09), Taishan Scholars Program (tsqn202507319), the New Cornerstone Science Foundation through the XPLORER PRIZE, and the Beijing Natural Science Foundation (QY24160).

## Conflicts of Interest

The authors declare no conflicts of interest.

## Supporting information




**Supporting File**: advs74737‐sup‐0001‐SuppMat.docx.

## Data Availability

The data that support the findings of this study are available from the corresponding author upon reasonable request.
