# Supplementary material for: Bioinspired Hierarchical Architecture with Water Transport Channels for Strong Adhesion at the Sweating Interface
Source: Adv Sci (Weinh). 2026 Apr 7;13(28):e24043. doi: 10.1002/advs.202524043 (PMC13185848; doi:10.1002/advs.202524043)
Supplement: Supplementary file 1 — Supporting File: advs74737‐sup‐0001‐SuppMat.docx. [file ADVS-13-e24043-s001.docx]

Supporting Information

**Bioinspired Hierarchical Architecture with Water Transport Channels for Strong Adhesion at the Sweating Interface**

*Jieliang Zhao^1,2^*^†^,* *Yu Xiang**^1†^, Heng Wang^1^, Huiqing Xu^1^, Zhong Liu^1^, Lulu Liang^1^, Fengwen Jiang^1^, Xuemei Chen^2^**, Wenzhong Wang^1^, Chenqing Liu^3^**, Guodong Liu^1^*, Shaoze Yan^4^*, Xu Hou^5^**

1. School of Mechanical Engineering, Beijing Institute of Technology, Beijing, 100081, P. R. China

2. Institute of Advanced Technology, Beijing Institute of Technology, Jinan 250300, China

3. College of Otolaryngology Head and Neck Surgery, Chinese PLA General Hospital, Beijing 100853, P. R. China

4. Department of Mechanical Engineering, Tsinghua University, Beijing, 100084, P. R. China.

5. Institute of Electrochemical Science and Engineering, State Key Laboratory of Physical Chemistry of Solid Surfaces, College of Chemistry and Chemical Engineering, Xiamen University, Xiamen 361005, P. R. China

^†^These authors contributed equally: Jieliang Zhao, Yu Xiang

*These authors jointly supervised this work: Jieliang Zhao, Guodong Liu, Shaoze Yan, Xu Hou

***Corresponding authors**

Jieliang Zhao: [jieliangzhao@bit.edu.cn](jielzhao@bit.edu.cn)

Guodong Liu: [liugd13@bit.edu.cn](https://d.docs.live.net/d48f1b13ab32d6a8/Master_1-1/1-Project/01-bionic%20permeable%20adhensive%20patch/0-手稿/补充材料修改/liugd13@bit.edu.cn)

Shaoze Yan: [yansz@tsinghua.edu.cn](mailto:yansz@tsinghua.edu.cn)

Xu Hou: [houx@xmu.edu.cn](mailto:houx@xmu.edu.cn)

**Supplementary notes**

Note S1. Calculation and Analysis of Multi-Stage Fluid Load-Bearing Volume in HT Textures

The triple structure of the HT texture corresponds to three liquid filling states. Under the minimum filling condition, the liquid fills the third-level cavity: the hexagonal groove at the top of the hexagonal prism. Neglecting geometric fillets, the volume of liquid that can be contained in each hexagonal prism is:

$$\begin{aligned} V_{3}=\cdot\frac{3\sqrt{3}}{2}\cdot hl^{2}\#\left( S1 \right) \end{aligned}$$

Each hexagonal prism features a hexagonal pit on top with a depth of h and a side length of *l*. The number of such pits is *n₃* = 49.

The second-level fluid-holding structure consists of groove networks between the hexagonal prisms, which can be further categorized into small grooves between adjacent prisms and large grooves between adhesive regions.

The drainage volume surrounding a single hexagon at this level can be expressed as:

$$\begin{aligned} V_{2-1}=\frac{3}{4}H\cdot W\cdot\left[ 2L+\left( 1+\frac{\sqrt{3}}{3} \right)W \right]\#\left( S2 \right) \end{aligned}$$

Here, *H* denotes the height of the hexagonal prisms, W the spacing between them, and *L* the side length. The number of hexagonal prisms in a single adhesive region is *n₂₋₁* = 165, while the number of hexagonal regions per unit area is *n₂₋₂* = 9.

Both the prisms and regions are regular hexagons, and the side length of the hexagonal region is 16 times that of a single prism, denoted as *M*. Accordingly, the relationship between their fluid-holding volumes is given by *V₂₋₂ = M³V₂₋₁*.

The first-level fluid-holding structure consists of conical drainage holes. In the volume calculation, the smaller grooves are neglected, and the structure is simplified as a truncated cone:

$$\begin{aligned} V_{1}=\frac{1}{3}\pi h_{r}\left( R_{1}^{2}+R_{1}R_{0}+R_{0}^{2} \right)\#\left( S3 \right) \end{aligned}$$

Here, *hᵣ* represents the channel height, while *R₁* and *R₀* denote the base and top diameters of the channel, respectively.

During the wet adhesion process, the liquid volume changes due to evaporation and drainage, and the total volume variation is represented as *V_total_*:

$$V_{1}+\left( V_{2-1}+V_{2-2} \right)+V_{3}\to V_{1}+\left( V_{2-1}+V_{2-2} \right)\to V_{1}$$

Based on the assumptions of volume conservation and geometric constraints, the critical height of the liquid bridge during wet adhesion is expressed as:

$$\begin{aligned} h_{crit}\approx\frac{V_{total}}{n{R_{l}}^{2}sin^{2}\theta}\#\left( S4 \right) \end{aligned}$$

The amount of liquid held at different structural stages corresponds to the size and number of the formed liquid bridges. In the equation, *h_crit_* denotes the critical height of the liquid bridge, *n* represents the number of unit structures capable of forming liquid bridges, *R* is the characteristic radius of the liquid bridge contact area—calculated based on the structure’s outer perimeter—and *θ* is the contact angle.

Note S2. Detailed Derivation of the Normal Liquid Bridge Force in HT Textures

During the wet adhesion process, the primary adhesive forces are the Laplace negative pressure and the tension acting along the boundary of the wetted area (the three-phase contact line). In the case of liquid bridges, these two forces are defined as follows[1]:

$$\begin{aligned} f_{1}=A\cdot\Delta P=A\cdot\frac{\gamma\left( cos\theta_{t}+cos\theta_{b} \right)}{h}\#\left( S5 \right) \end{aligned}$$

$$\begin{aligned} f_{2}=\gamma\cdot l\#\left( S6 \right) \end{aligned}$$

In the equation, *γ* represents the liquid surface tension, and *A* is the area of the solid surface wetted by the liquid. *h* denotes the distance between the textured surface and the contact surface. For an ideal liquid bridge with spherical side surfaces, the liquid bridge height satisfies the geometric relationship *h = r₁ (cos θ_P_ + cos θ_S_)*, where *θ_P_* and *θ_S_* are the contact angles on the texture side and the contact surface side, respectively.*l* is the length of the three-phase contact line. In the liquid bridge model, the upper and lower surfaces form an action-reaction force pair. Therefore, for the entire system, the total liquid bridge force can be represented by considering only one end, expressed as[2,3]:

$$\begin{aligned} f_{cap}=f_{1}+f_{2}=A\cdot\frac{\gamma\left( cos\theta_{t}+cos\theta_{b} \right)}{h}+\gamma l\#\left( S7 \right) \end{aligned}$$

During contact, HTP textures form liquid bridges, whose number and size vary continuously with changes in the liquid film. The capillary force generated between the HTP texture and the contacting surface is denoted as *F_cap1_*. Meanwhile, drainage grooves of certain depth on the HTP texture create new menisci during separation, producing an additional capillary force *F_cap2_*. These two force components can be expressed as[4]:

$$\begin{aligned} F_{cap}^{1}=n_{1}\left[ \frac{\gamma\cdot\left( cos\theta_{p}+cos\theta_{b} \right)}{h}\cdot A_{h}+l_{h}\cdot\gamma cos\theta_{p} \right]\#\left( S8 \right) \end{aligned}$$

$$\begin{aligned} F_{cap}^{2}=n_{2}\left( \frac{2\gamma\cdot cos\theta_{p}}{W}\cdot A_{w}+l_{w}\cdot\gamma cos\theta_{p} \right)\#\left( S9 \right) \end{aligned}$$

Due to the inherent fractal characteristics of the HTP structure, the three-stage adhesion based on liquid volume conservation follows a similar adhesion law. The total normal adhesive force is expressed as:

$$\begin{aligned} F_{n}=F_{cap}^{1}+F_{cap}^{2}\#\left( S10 \right) \end{aligned}$$

Here, *nᵢ* corresponds to the number of liquid bridges formed, *A_h_* is the area of a single liquid bridge, and *l_h_* is the perimeter of the liquid bridge near the texture surface. In the second part, *n_i+1_*denotes the number of grooves, which equals the number of liquid bridges formed in the subsequent stage. *W* is the groove width, *A_b_* is the horizontal projected area of the groove, and *l_w_* represents the three-phase contact line length at the groove (solid-liquid-gas interface).

Furthermore, to investigate the proportion of capillary forces contributed by the liquid bridge and the meniscus within the grooves to the total normal force, the capillary force ratio can be expressed as:

$$\begin{aligned} Rt_{cap1}=\frac{F_{cap}^{1}}{F_{cap}^{1}+F_{cap}^{2}} \\ =\frac{n_{i}\left[ \frac{\gamma\cdot\left( cos\theta_{p}+cos\theta_{b} \right)}{h_{crit}}\cdot A_{h}+\gamma\cdot l_{h}cos\theta_{p} \right]}{n_{i}\left[ \frac{\gamma\cdot\left( cos\theta_{p}+cos\theta_{b} \right)}{h_{crit}}\cdot A_{h}+\gamma\cdot l_{h}cos\theta_{p} \right]+n_{i+1}\left( \frac{2\gamma\cdot cos\theta_{p}}{W}\cdot A_{W}+\gamma\cdot l_{W}cos\theta_{p} \right)}\#\left( S11 \right) \end{aligned}$$

When the contact angles of the textured surface and the contacting plane are close or equal, i.e., *θ_P_ ≈ θ_S_*, a further simplified expression for the capillary force ratio can be derived as:

$$\begin{aligned} Rt_{cap1}=\frac{F_{cap}^{1}}{F_{cap}^{1}+F_{cap}^{2}} =\left[ 1+\frac{n_{i+1}\left( \frac{A_{w}}{W}+l_{w} \right)}{n_{i}\left( \frac{A_{h}}{\text{h}_{\text{crit}}}+l_{h} \right)} \right]^{-1}\#\left( S12 \right) \end{aligned}$$

Here, the characteristic length governing the dominant capillary force can be expressed as $\kappa=n\left( \frac{A}{d}+l \right)$, That is, during the formation of multi-level continuous capillarity, the number of menisci, the ratio of projected area to gap size, and the contact length together determine the magnitude and proportion of the capillary force. This insight provides valuable guidance for the regulation of capillary forces.

As evaporation and structure-guided drainage progress, the liquid volume between the HTP texture and the substrate decreases accordingly. Notably, when the liquid bridge height *h* ＜0.5 mm, liquid enters the adhesive surface from the cavity grooves, resulting in the formation of gas cavities between the hexagonal framework, the substrate, and the liquid film. Menisci form on both sides of the gas cavity with unequal curvatures, generating a pressure difference expressed as:

$$\begin{aligned} \delta=\Delta P_{right}-\Delta P_{left}=\frac{\gamma}{h}\left[ \left( cos\theta_{ft}+cos\theta_{fb} \right)-\left( cos\theta_{rt}+cos\theta_{rb} \right) \right]\#\left( S13 \right) \end{aligned}$$

Here, *θ* represents the advancing and receding contact angles of the textured surface and the substrate. Under these conditions, the tangential adhesive force of the HTP texture consists of friction induced by the normal force (with the friction coefficient defined as *μ*) and the pinning force caused by the gas cavity, which can be expressed as:

$$\begin{aligned} F_{s}=\mu\cdot F_{n}+F_{af}\#(S14) \end{aligned}$$

The pinning force is expressed as:

$$\begin{aligned} F_{af}=N_{1}N_{2}\sum_{n=1}^{N_{i}N_{2}} n\cdot\delta\cdot S_{\delta}=\frac{N_{1}^{2}N_{2}^{2}\left( N_{1}N_{2}+1 \right)\delta\cdot S_{\delta}}{2}\#\left( S15 \right) \end{aligned}$$

In the equation, *N_1_*, *N_2_*represent the number of gas cavities extending along the orthogonal directions centered on the hexagon. For simplification, the quantities along one direction are denoted as *n₁*, and along the orthogonal direction as *n₂*, with approximations $N_{1}\approx\sqrt{n_{1}},N_{2}\approx\sqrt{n_{2}}$. *S_δ_* is the cross-sectional area in the direction of the liquid bridge height.The tangential force enhancement factor e caused by the cavities is expressed as:

$$\begin{aligned} e=\frac{\mu\cdot F_{n}+F_{af}}{\mu\cdot F_{n}}=1+\frac{F_{af}}{\mu\cdot F_{n}}\#\left( S16 \right) \end{aligned}$$

Note S3. Principles of Numerical Simulation Analysis for Vertical Capillary Filling in Conical Channels

In COMSOL finite element analysis, the level set method is employed to formulate the equations[5]. The fluid interface is represented by the level set function $\boldsymbol{\phi}\boldsymbol{=}\boldsymbol{0.5}$, where $\boldsymbol{\phi}\boldsymbol{s=0}$ air and $\boldsymbol{\phi}\boldsymbol{=1}$ in water. Thus, the level set function can be interpreted as the volume fraction of water. The interface transport between the two fluid phases is governed by the following equation:

$$\begin{aligned} \frac{\partial\phi}{\partial t}+u\cdot\nabla\phi=\gamma\nabla\cdot\left( \varepsilon\nabla\phi-\phi\left( 1-\phi\right)\frac{\nabla\phi}{\left| \nabla\phi\right|} \right)\#\left( S17 \right) \end{aligned}$$

The parameter$\varepsilon$determines the thickness of the interface. When numerical stabilization methods are applied to the level set equation, the interface thickness is usually specified as $\varepsilon=\frac{h}{2}$*,* where h is the characteristic mesh size in the interface region. The parameterγ determines the amount of reinitialization, and an appropriate value of *γ* is the maximum velocity magnitude appearing in the model. The density and viscosity in the multiphysics coupling are defined by the following formulas:

$$\begin{aligned} \rho=\rho_{air}+\left( \rho_{water}-\rho_{air} \right)\phi\#\left( S18 \right) \end{aligned}$$

$$\begin{aligned} \mu=\mu_{air}+\left( \mu_{water}-\mu_{air} \right)\phi\#\left( S19 \right) \end{aligned}$$

According to the above definitions, the density and viscosity coefficients vary smoothly across the fluid interface. The 𝛿 function can be approximated as:

$$\begin{aligned} \delta=6\left| \phi\left( 1-\phi\right) \right|\left| \nabla\phi\right|\#\left( S20 \right) \end{aligned}$$

The interface normal vector is calculated according to the following expression:

$$\begin{aligned} n=\frac{\nabla\phi}{\left| \nabla\phi\right|}\#\left( S21 \right) \end{aligned}$$

The model simulates fluid mass and momentum transfer based on the incompressible Navier–Stokes equations. To account for the effects of surface tension, surface tension must be included in the model. Therefore, the Navier–Stokes equations are given by:

$$\begin{aligned} \rho\frac{\partial u}{\partial t}+\rho\left( u\cdot\nabla\right)u=\nabla\cdot\left[ -pI+\mu\left. \nabla u+\left( \nabla u \right)^{T} \right. \right]+F_{st}+\rho g\#\left( S22 \right) \end{aligned}$$

$$\begin{aligned} \nabla\cdot u=0\#\left( S23 \right) \end{aligned}$$

Here, *ρ* represents density (kg/m³), *μ* is the dynamic viscosity (Ns/m²), *u* denotes velocity (m/s), *p* is pressure (Pa), *g* is the gravitational acceleration vector (m/s²), and *F_st ​_* is the surface tension force acting at the air/water interface.The surface tension in the simulated level set interface is calculated as:

$$\begin{aligned} F_{st}=\sigma\delta\kappa n\#\left( S24 \right) \end{aligned}$$

Here, *n* is the interface normal vector, 𝜎 is the surface tension coefficient (N/m), $\kappa=-\nabla\cdot n$ is the curvature, and 𝛿 is the Dirac delta function, which is nonzero only at the fluid interface.

The following boundary force is added to enforce the contact angle

$$\begin{aligned} F_{\theta}=\sigma\delta\left( n_{wall}\cdot n-cos\theta_{w} \right)n\#\left( S25 \right) \end{aligned}$$

Here, 𝜃 is the contact angle. If a no-slip boundary condition is applied, the velocity at that boundary becomes zero, making it impossible to specify the contact angle, while the interface remains pinned to the wall. However, if a small amount of slip is allowed, the contact angle can be specified. The 'Wetted Wall' coupling feature adds terms given by the boundary force equation, thereby enabling the setting of the contact angle.

The 'Wetted Wall' feature applies to solid walls in contact with fluid–fluid interfaces. In the 'Level Set' case, it is introduced as a multiphysics coupling feature that sets the velocity component normal to the wall to zero; that is:

$$\begin{aligned} u\cdot n_{wall}=0\#\left( S26 \right) \end{aligned}$$

The added boundary friction force is:

$$\begin{aligned} F_{\mathrm{fr}}=-\frac{\mu}{\beta}u\#\left( S27 \right) \end{aligned}$$

Here, 𝛽 is the slip length, which is equal to the mesh element size ℎ at the wall-water-air interface.

Note S4. Detailed derivation of the forces driving liquid rise in conical channels by Laplace pressure

The primary driving force for unidirectional flow through the conical pore is the Laplace pressure difference, which is defined for the spherical meniscus in this problem as[6]:

$$\begin{aligned} \Delta P=P_{gas}-P_{liquid}=\frac{1}{r}\cdot2\gamma\#\left( S28 \right) \end{aligned}$$

The pressure difference is the gas-side pressure minus the liquid-side pressure. 𝑟 is the radius of the meniscus, which, according to geometric relations, satisfies:

$$\begin{aligned} \Delta P=\frac{2\gamma\cdot cos\left( \theta-\alpha\right)}{R_{1}}\#\left( S29 \right) \end{aligned}$$

Here, 𝑅_1_ is the height at the contact point of the meniscus.

$$\begin{aligned} F_{L}=\Delta P\cdot S=\frac{2\gamma\cdot\cos\left( \theta-\alpha\right)}{R_{1}}\cdot\pi R_{1}^{2}=2\pi R\gamma\cdot\cos\left( \theta-\alpha\right)\#\left( S30 \right) \end{aligned}$$

Here, 𝑅 is the channel radius corresponding to the height of the meniscus contact point.

The liquid volume in the scale of this study is on the milliliter level; therefore, the effects of gravity are not neglected. Gravity calculations are based on volume conservation among the channel, liquid phase, and gas phase, expressed respectively as:

$$\begin{aligned} V_{channel}=\frac{1}{3}\pi R_{2}^{3}\cdot\frac{R_{2}}{tan\alpha}=\frac{1}{3tan\alpha}\cdot\pi R_{2}^{4}\#\left( S31 \right) \end{aligned}$$

$$\left\{ \begin{aligned} V_{Air1}=\frac{1}{3}\pi R_{1}^{3}\cdot(\frac{R_{2}}{tan\alpha}-H)\#(S32) \\ V_{Air2}=\frac{\pi}{6}\cdot(H-\frac{R_{2}-R_{1}}{tan\alpha})\cdot[3\cdot(\frac{R_{1}}{cos(\theta-\alpha)})^{3}+(\frac{H-R_{2}-R_{1}^{2}}{tan\alpha})]\#(S33)\#\#\#\# \end{aligned} \right.$$

$$\begin{aligned} V_{liquid}=V_{channel}-\left( V_{Air1}+V_{Air2} \right)\#\left( S34 \right) \end{aligned}$$

The driving equation for the resultant force of liquid rise can be obtained as:

$$\begin{aligned} F=F_{L}-\rho V_{liquid}=2\pi R\gamma\cdot cos\left( \theta-\alpha\right)-\rho V_{liquid}\#\left( S35 \right) \end{aligned}$$

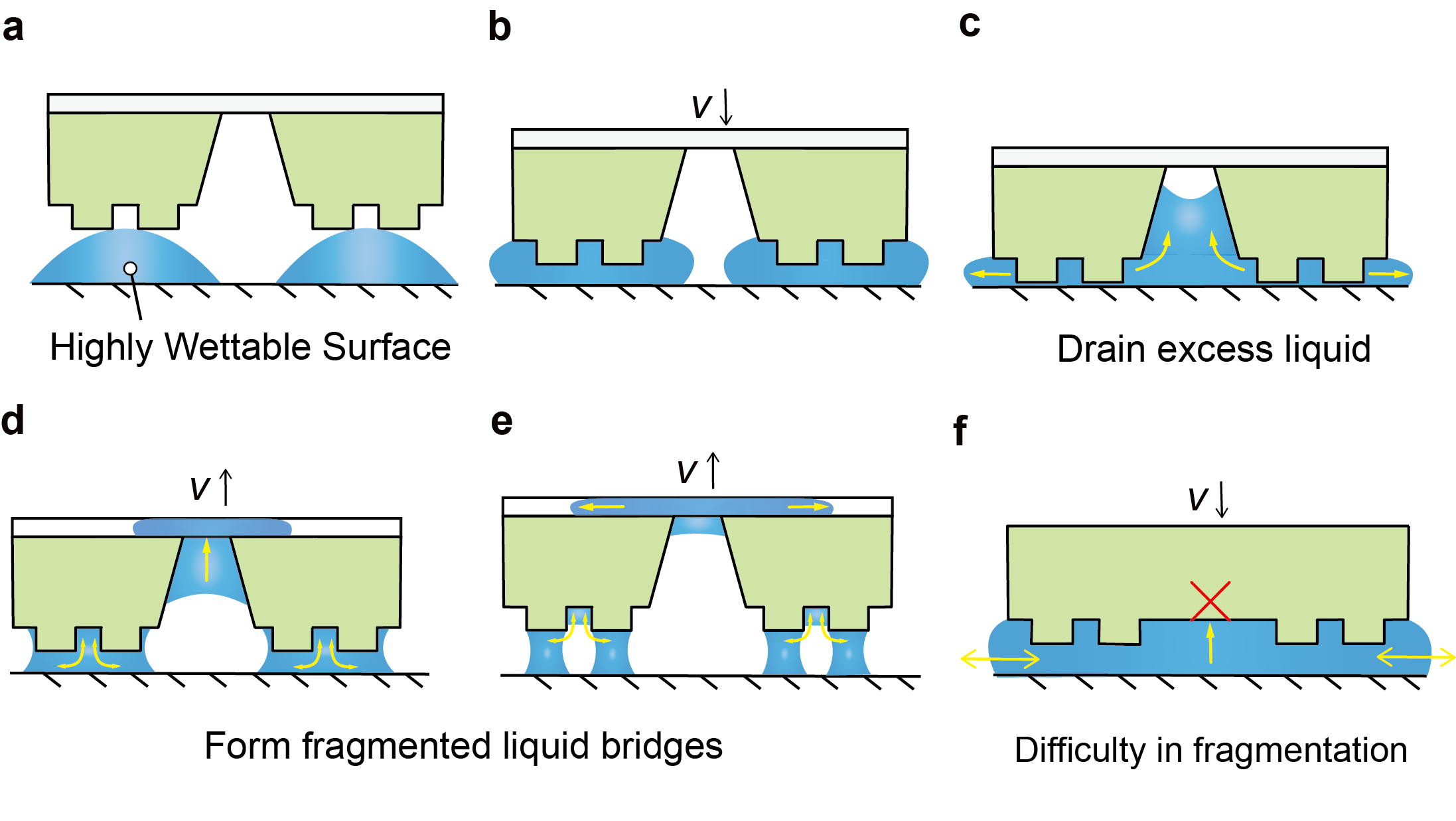


**Figure S1.** Comparison of drainage behavior on highly hydrophilic surfaces between patches with drainage structures and those without. **a–e** Patches with drainage structures generate fragmented liquid bridges through drainage when approaching highly hydrophilic surfaces. **f** Patches without drainage structures fail to effectively drain liquid upon approaching the surface, thus unable to form fragmented liquid bridges or enhance adhesion.


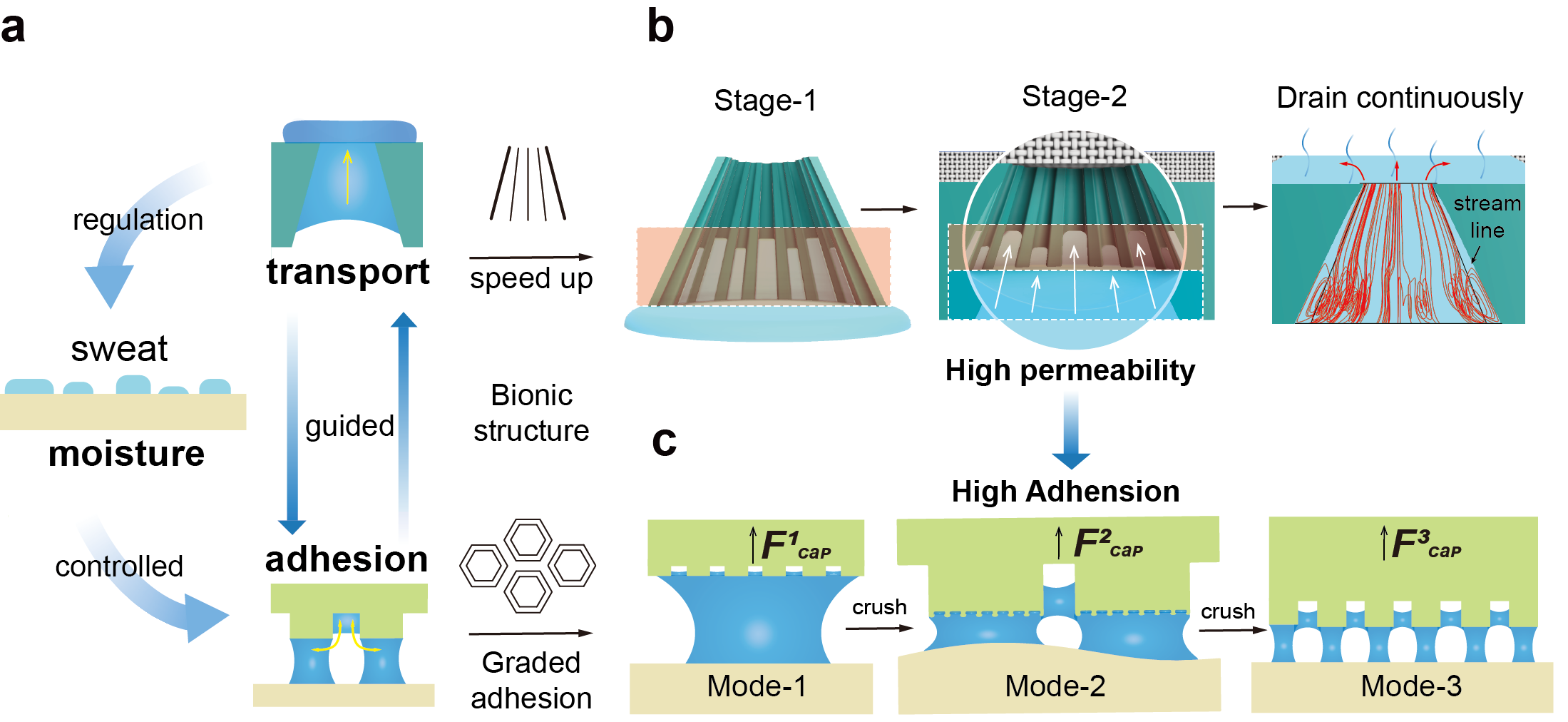


**Figure S2 .** The composite structure of waterfowl-pitcher plant water transport structure and bee-tree frog adhesion structure. **a** Water is used as the medium between the two structures to control adhesion and liquid transport. **b** The multi-groove conical channel completes liquid transport in a two-stage manner. **c** The liquid bridge adapts to adhesion states under various liquid volumes through three-stage fragmentation.


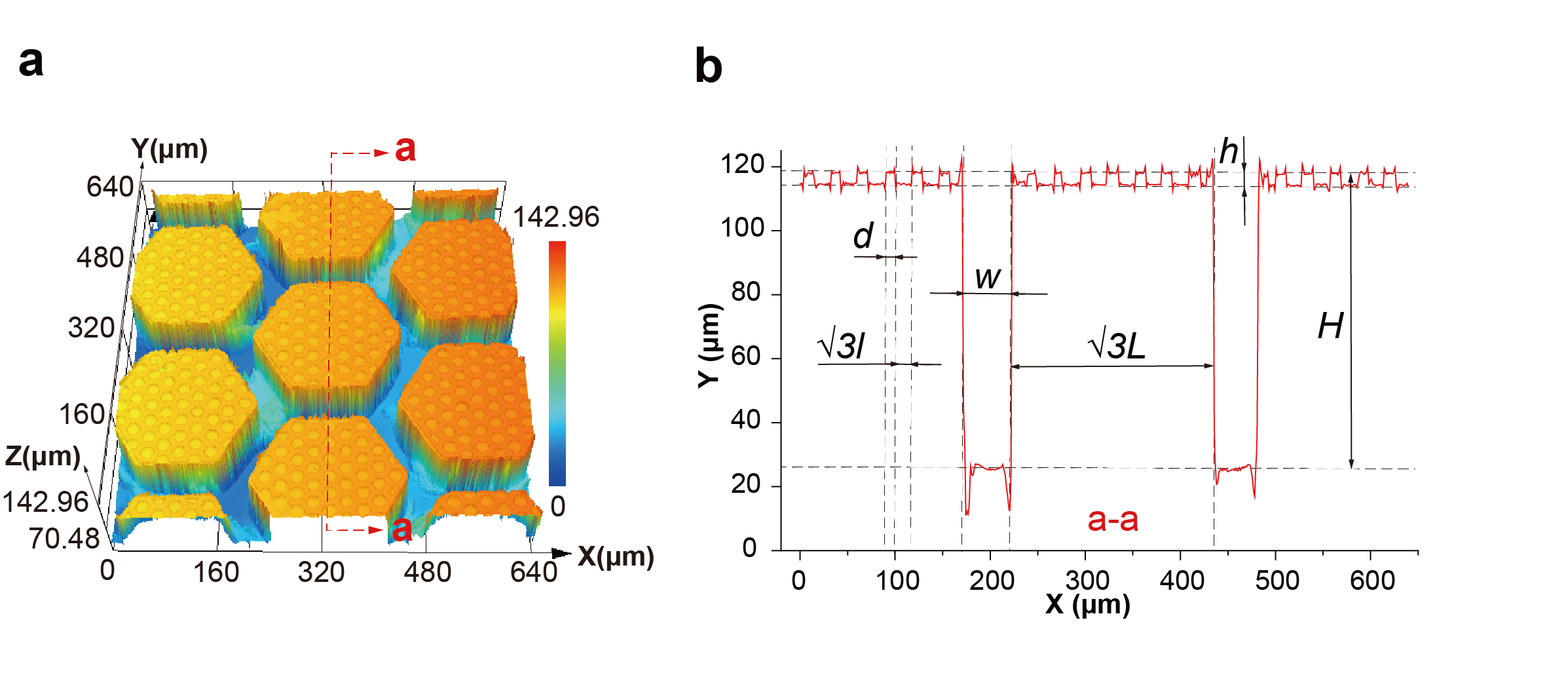


**Figure S3.** Confocal laser scanning microscopy (CLSM) image and corresponding profile curve of the HTP texture.


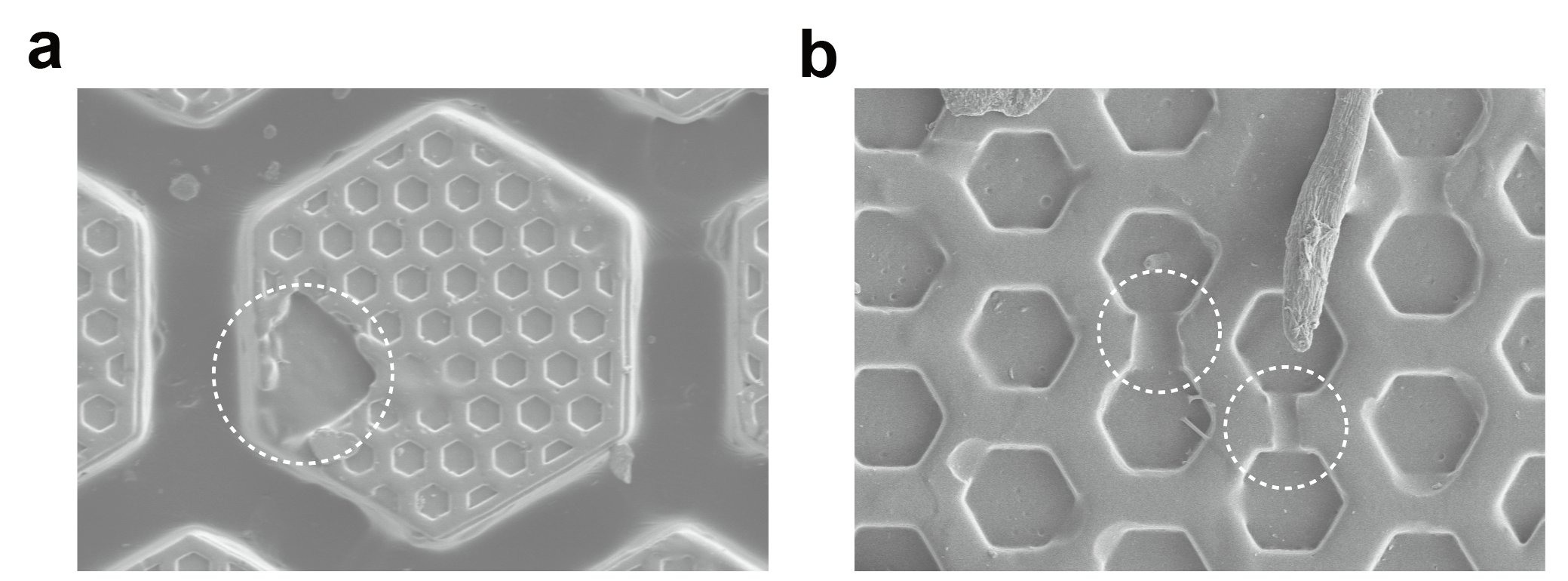


**Figure S4.** Surface morphology after 1000 peeling cycles. **a** Localized texture detachment (dotted circle) on the otherwise intact pillar. **b** Minor defects connecting adjacent micro-dimples. The damage is strictly superficial, confirming the absence of structural buckling.


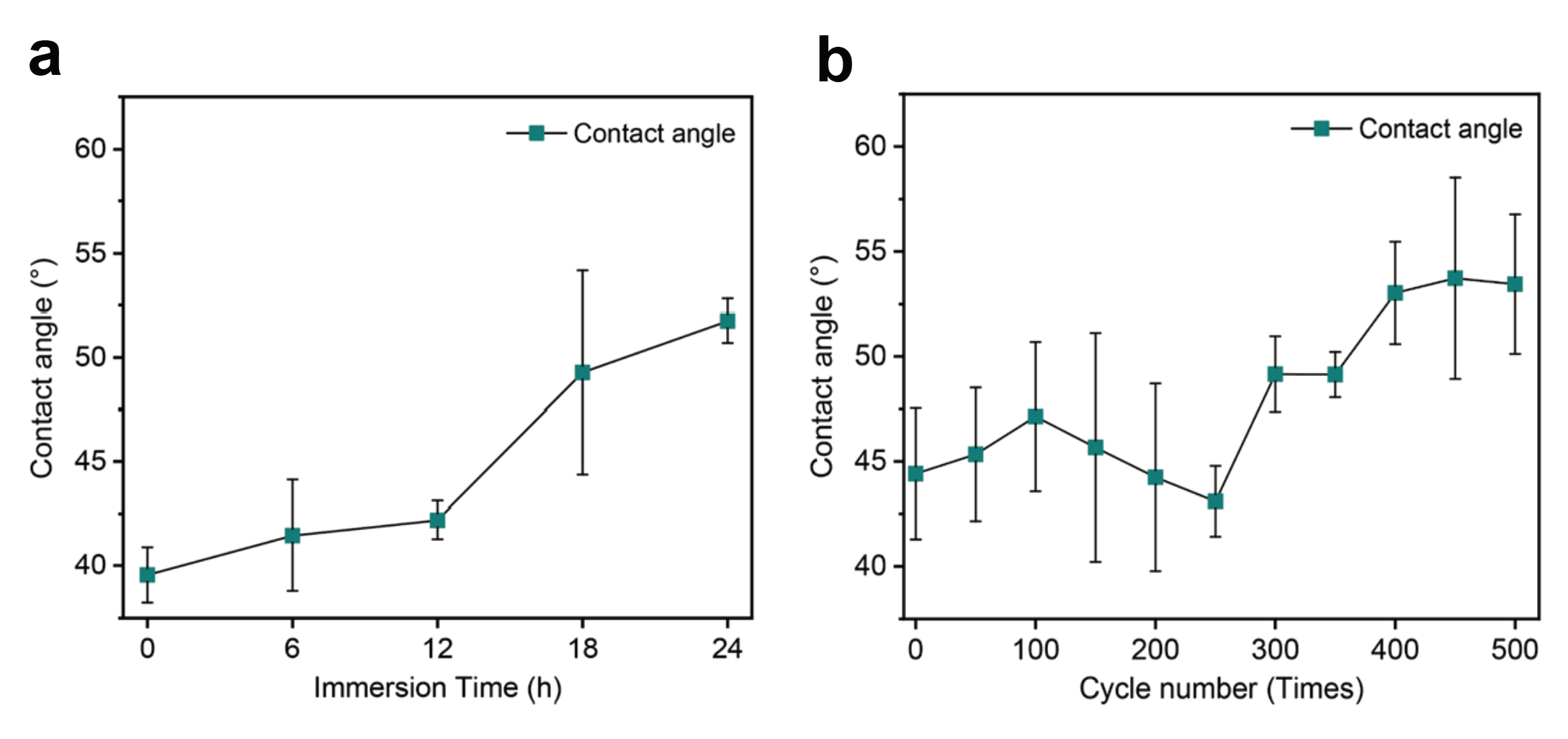
 **Figure S5.** Evaluation of the hydrophilic coating stability against sweat immersion and mechanical fatigue. **a** Contact angle changes over 24 hours of sweat immersion. **b** Contact angle fluctuation during 500 bending cycles.


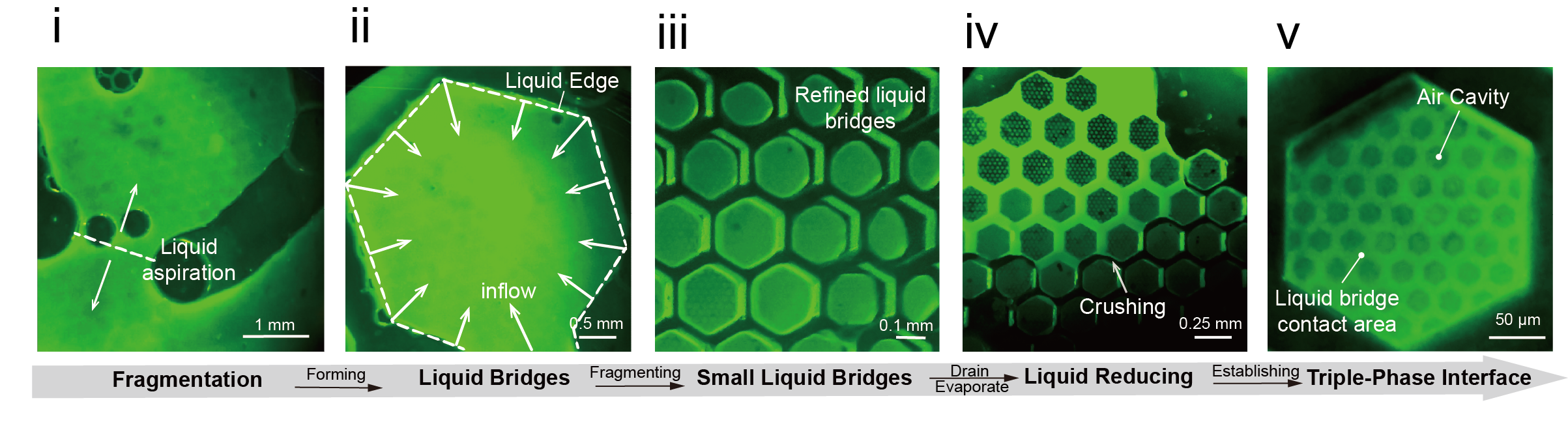


**Figure S6.** Evolution of liquid film morphology on the HTP texture visualized by fluorescent green dye diluted in deionized water.


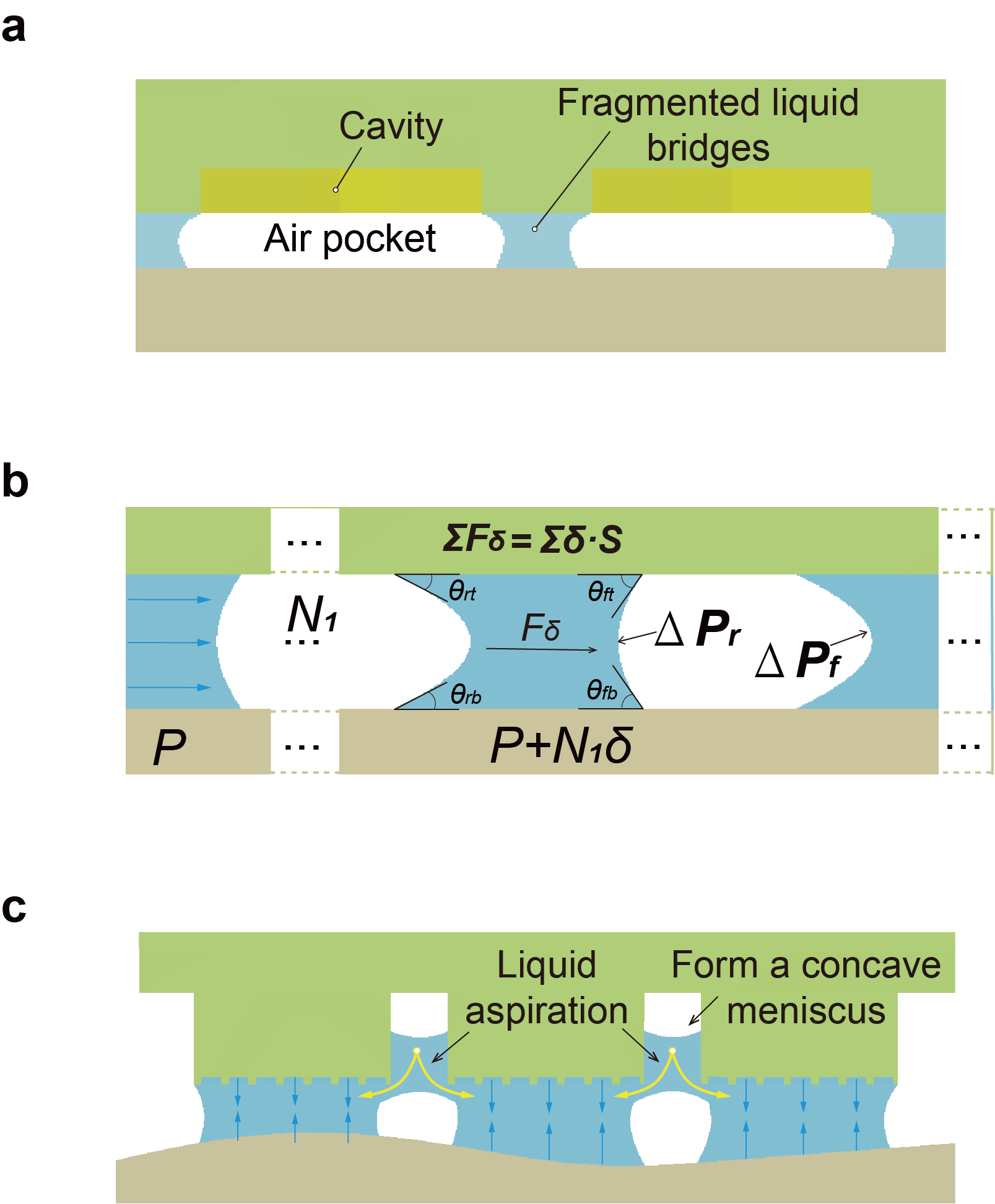


**Figure S7.** Mechanism of enhanced shear adhesion by HT texture-induced embolism effect and liquid migration mechanism: **a** Microstructures can induce the formation of air cavities, leading to the fragmentation of liquid bridges. **b** When a curvature difference between the left and right liquid surfaces is formed, it induces a pressure difference, which helps enhance the shear resistance. **c** During the structural displacement, liquid components can adaptively migrate from the grooves to the large liquid bridges.


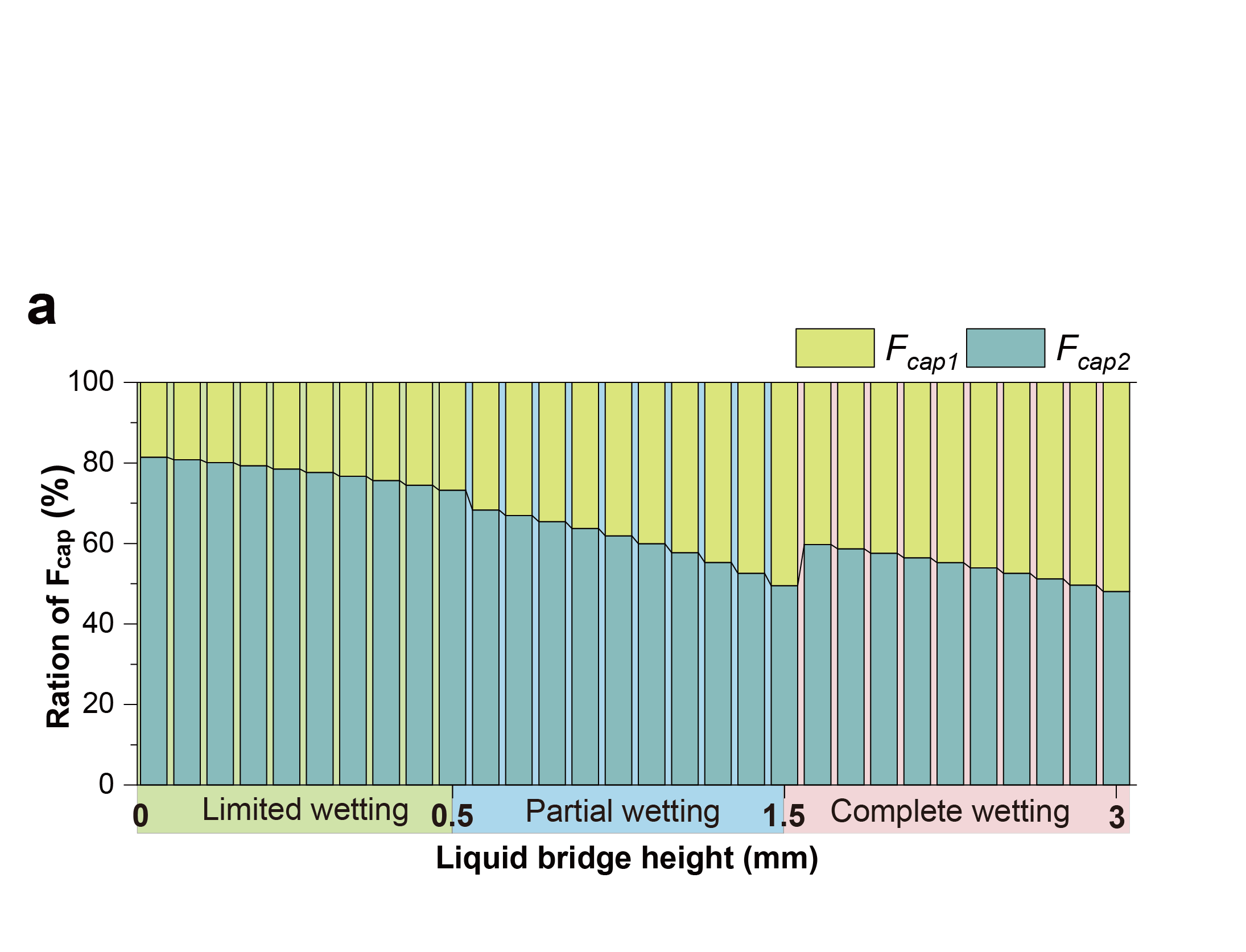


**Figure S8.** The proportion of the capillary force component in the three wetting stages.


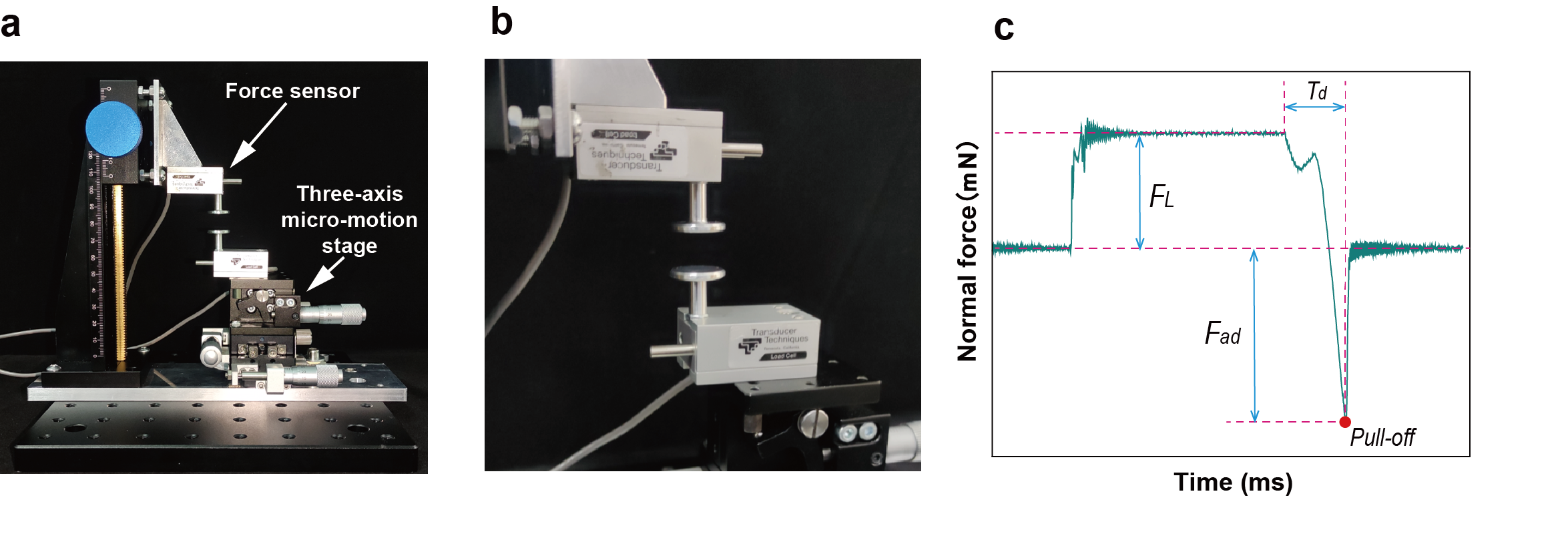


**Figure S9.** Adhesion force measurement setup: **a** Composed of an XYZ micro-positioning stage and a high-sensitivity force sensor. **b** Force sensor and extended test platform connector.


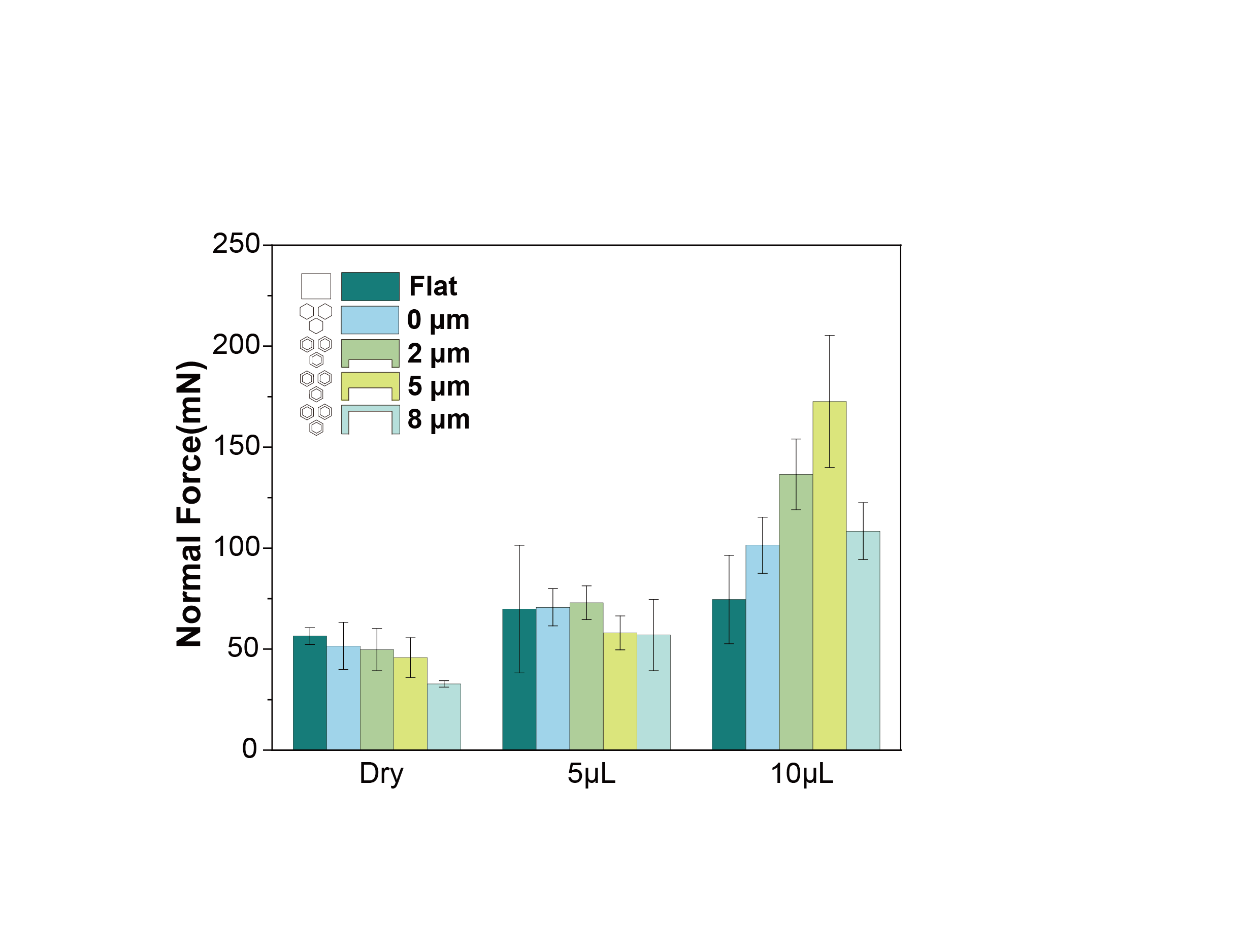


**Figure S10.** Normal adhesion performance of textures with different sizes under various wetting conditions.


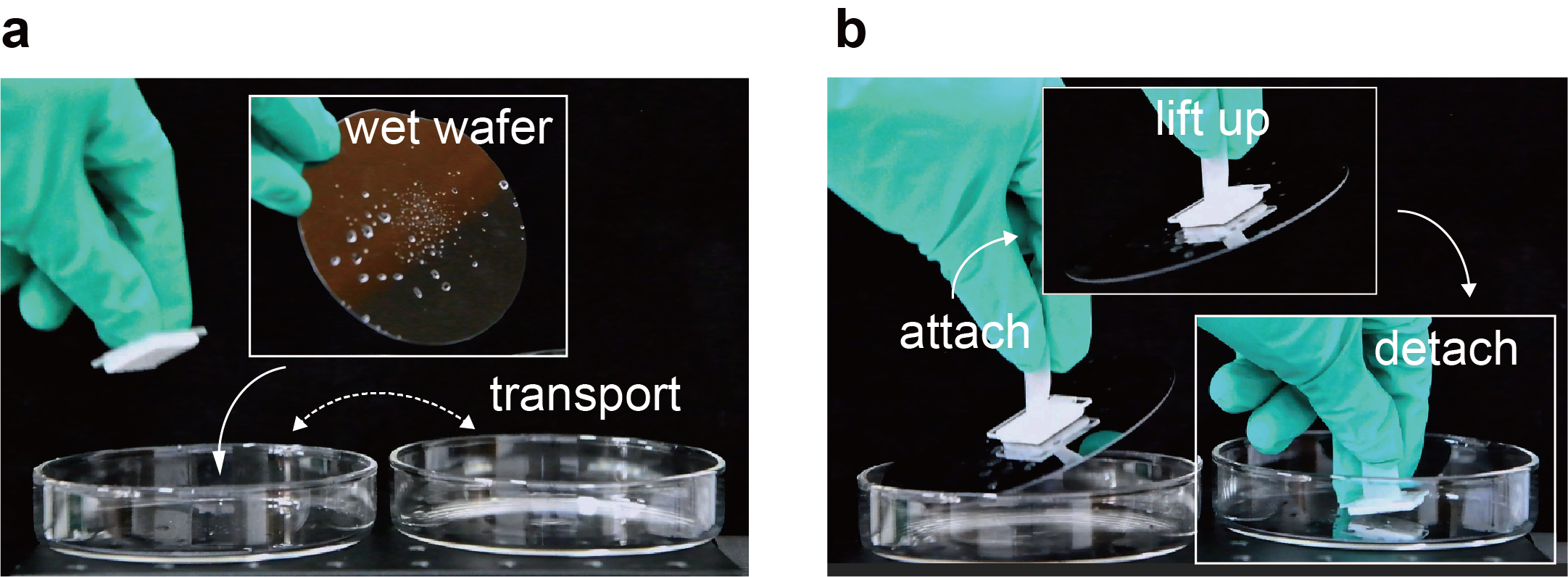


**Figure S11.** CMAP adhering to and transporting a moist silicon wafer.


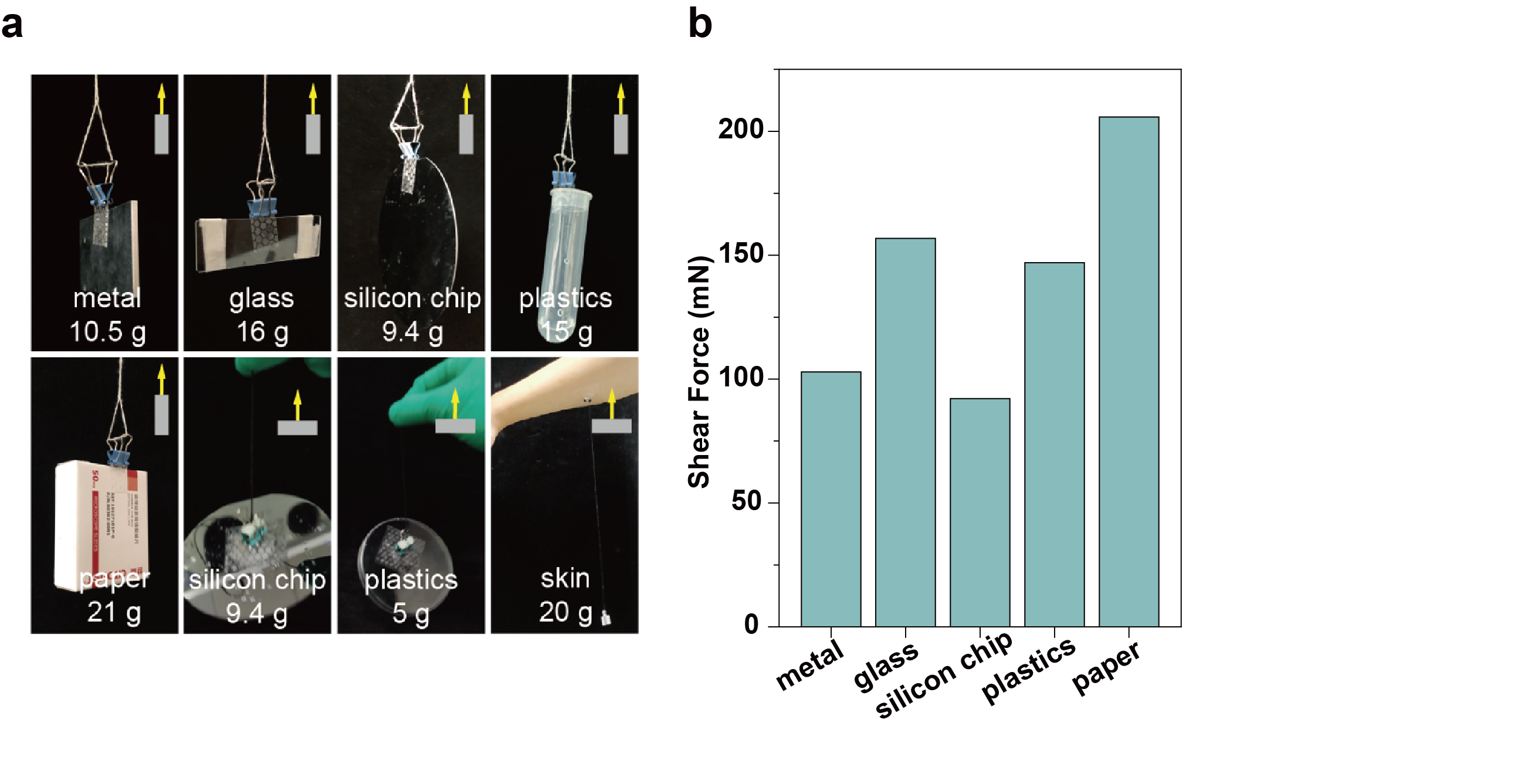


**Figure S12.** Measuring the force magnitude when lifting different surfaces with patches using HTP texture. **a** Normal and tangential load testing of CMAP patches on objects of different materials. **b** Tangential load and shear force measurements of CMAP patches holding different material samples


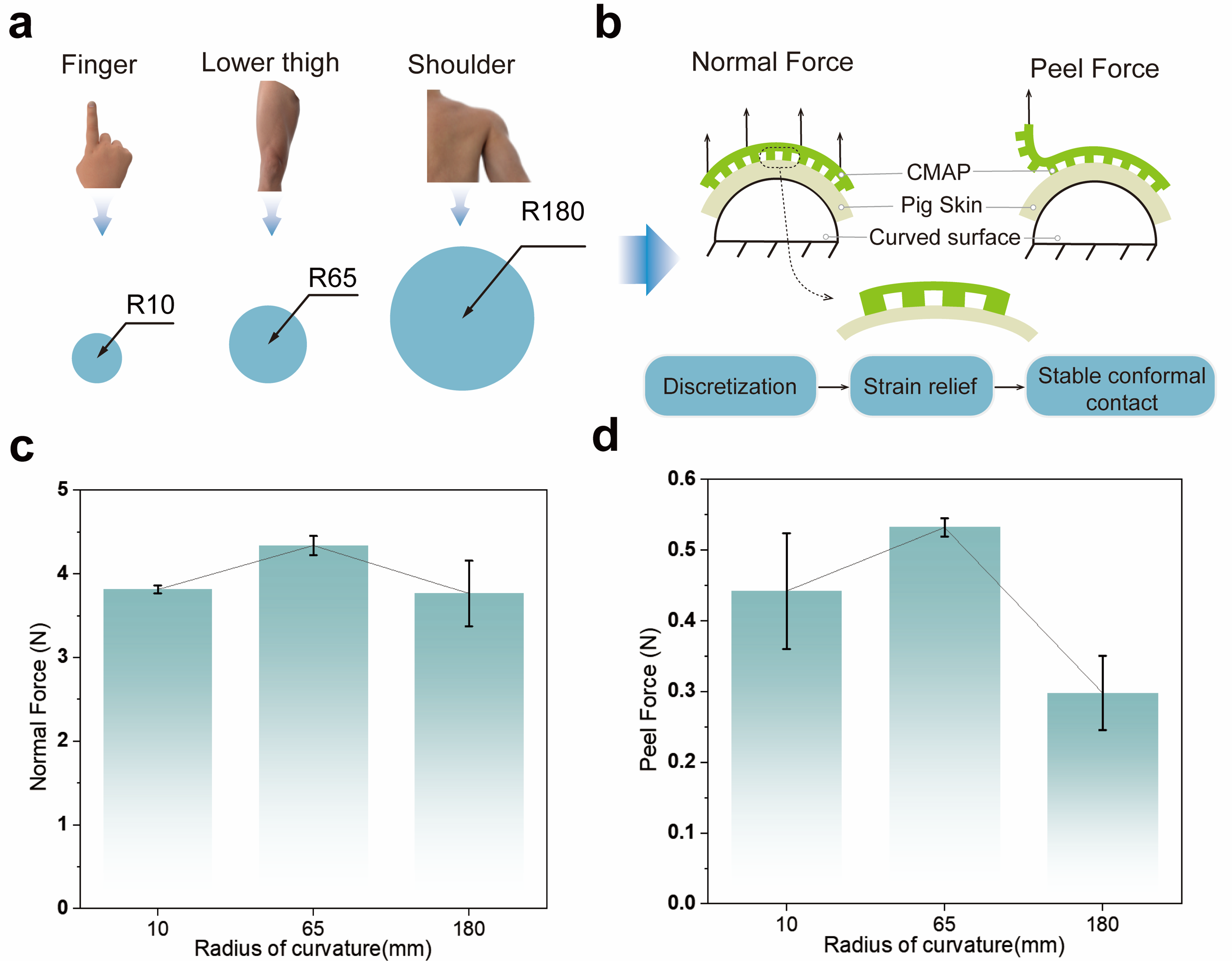


**Figure S13.** Adhesion adaptability on curved biological interfaces. **a** Curvature radii mimicking the finger (*R*=10 mm), lower thigh (*R*=65 mm), and shoulder (*R*=180 mm), derived from the 2012 Anthropometric Survey of U.S. Army Personnel. **b** Schematic showing strain relief and conformal contact on curved porcine skin enabled by the discretized structure. **c, d** Normal (c) and peel (d) adhesion forces on porcine skin across these curvatures, demonstrating robust stability (n=5).


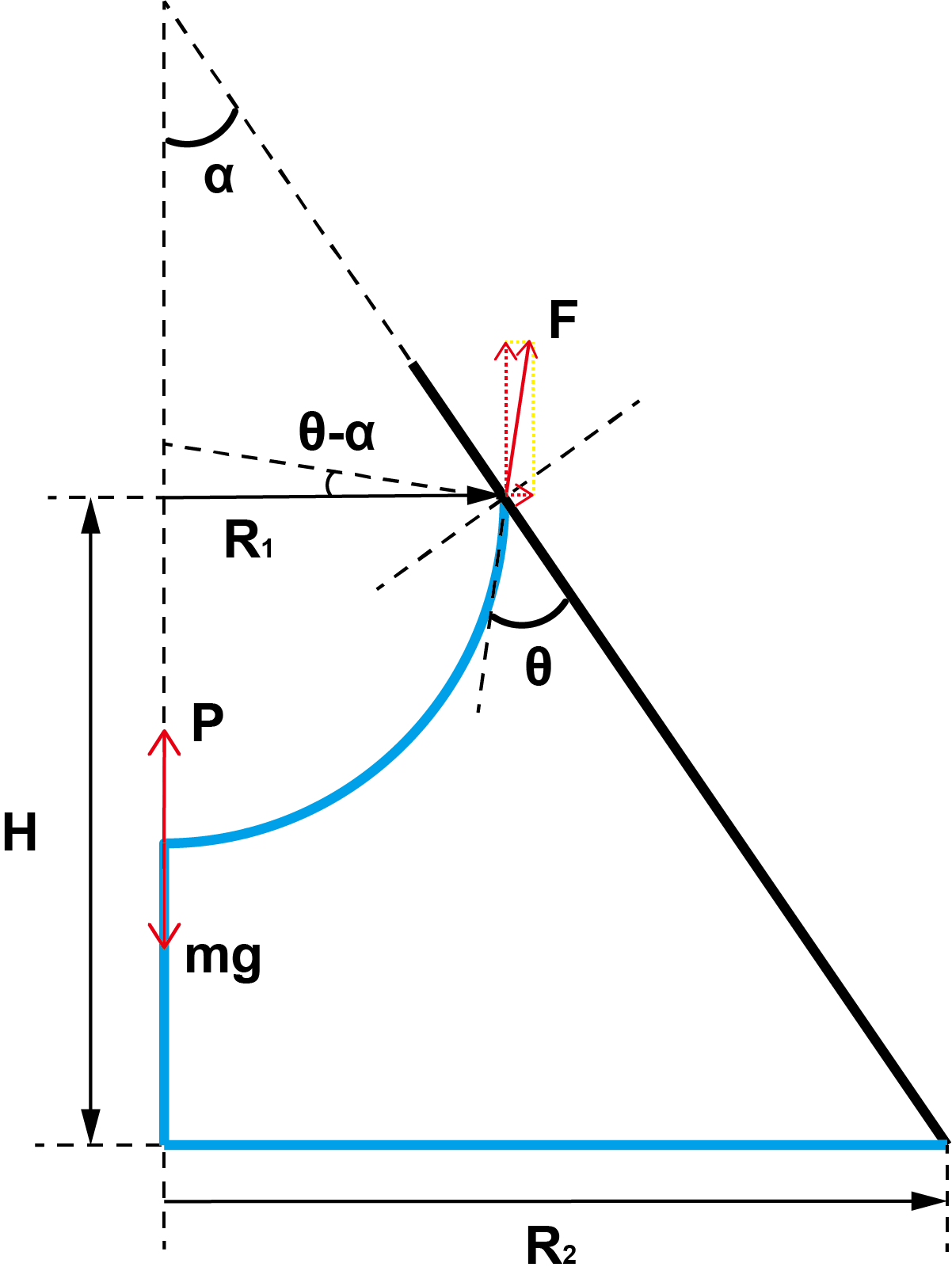


**Figure S14.** Geometrical and dynamic analysis of liquid rise in conical channels


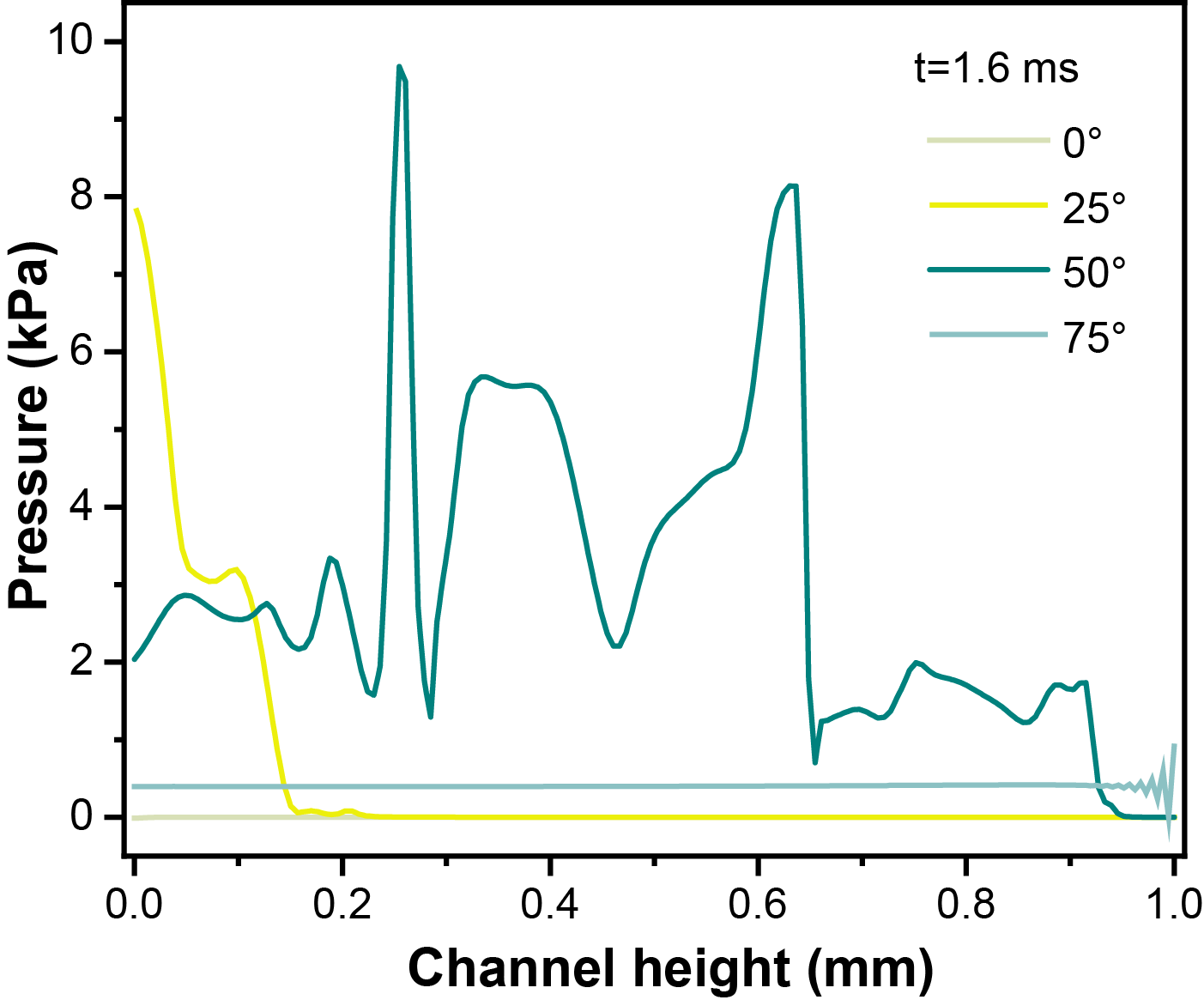


**Figure S15.** Pressure distribution along the axial direction of liquid rise in capillaries with different cone angles at 1.6 ms.


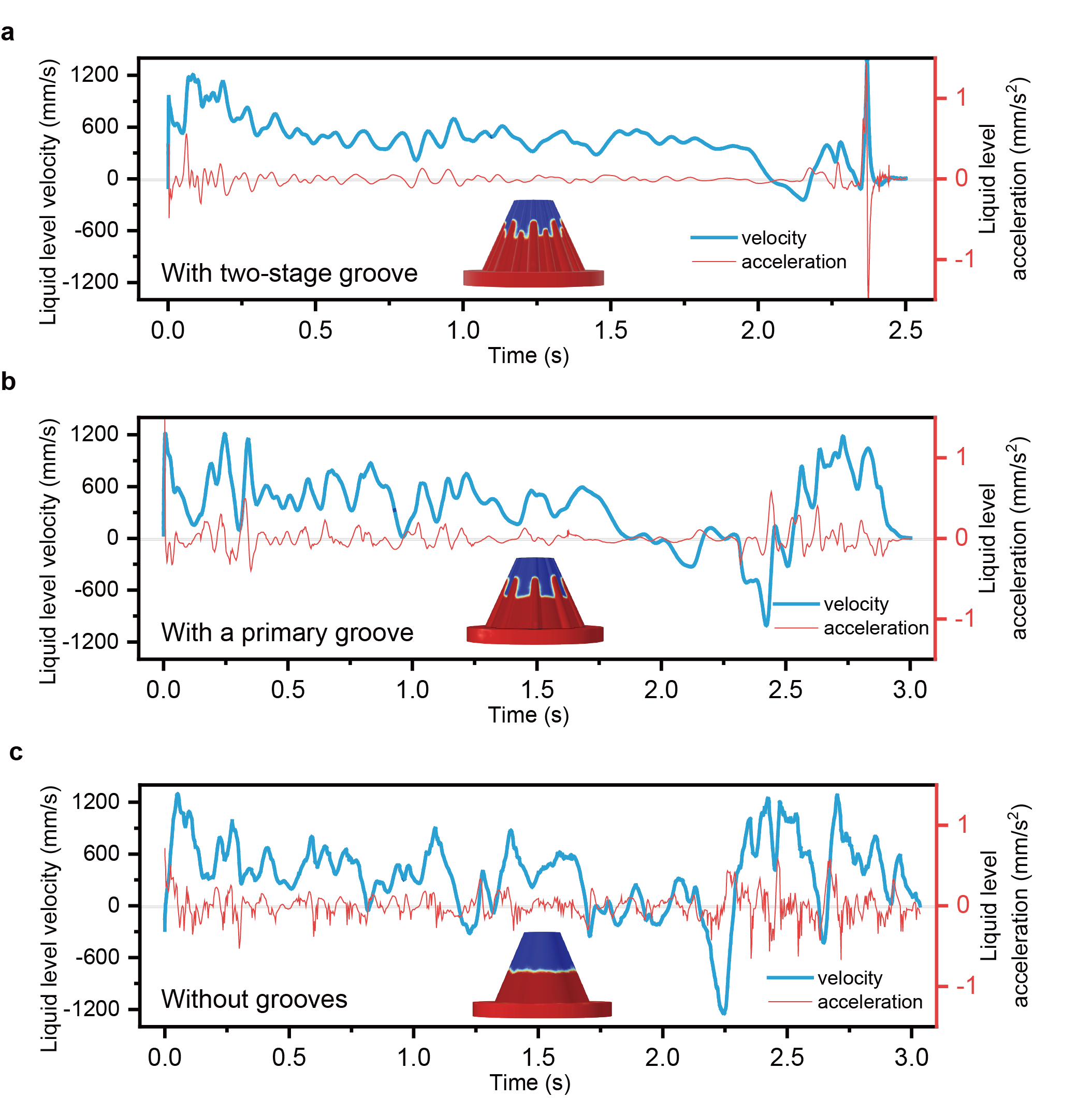


**Figure S16.** Velocity and acceleration curves of the solid–liquid–gas contact line rising in conical channels with different surface structures. Microgrooves help maintain stable velocity and acceleration, reducing negative fluctuations.


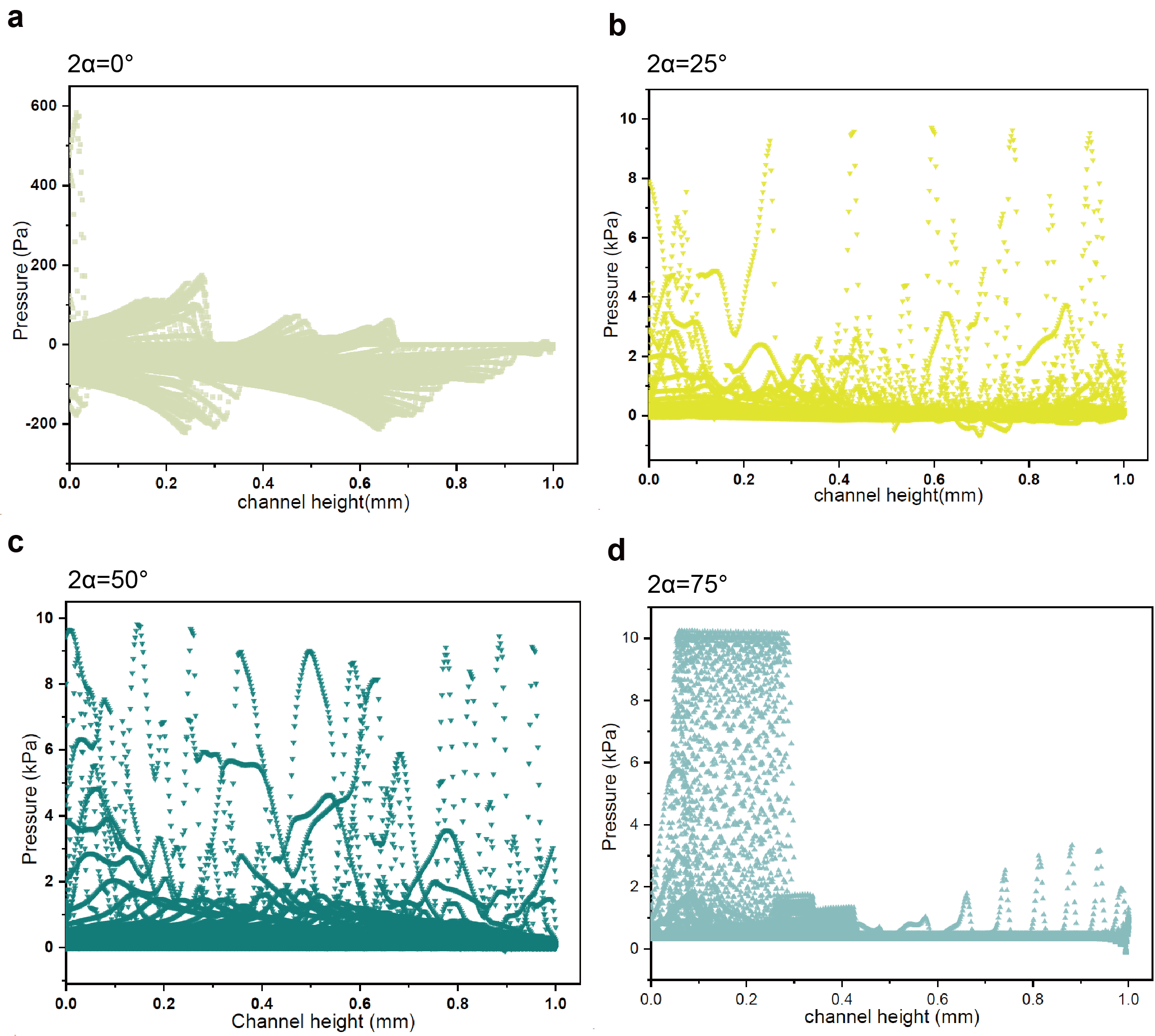


**Figure S17.** Pressure–time distribution along the central axis of conical capillary channels with cone angles of 0°, 25°, 50°, and 75°, plotted against height.At a cone angle of 50°, higher pressure peaks occur more frequently and are sustained longer, effectively driving liquid rise.


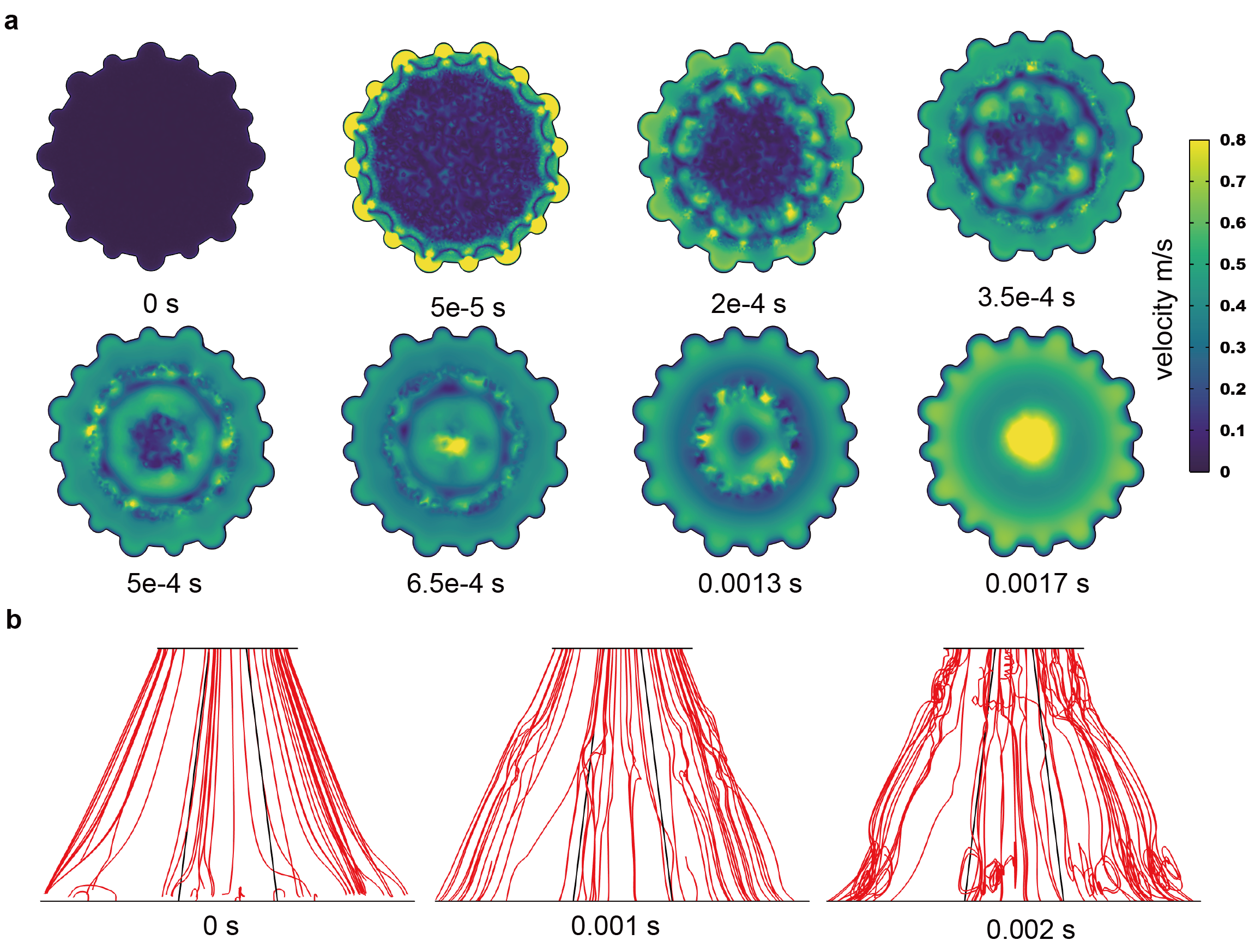


**Figure S18.** Velocity contour map at cross-section C–C and side-view streamline diagram in the actual conical channel.


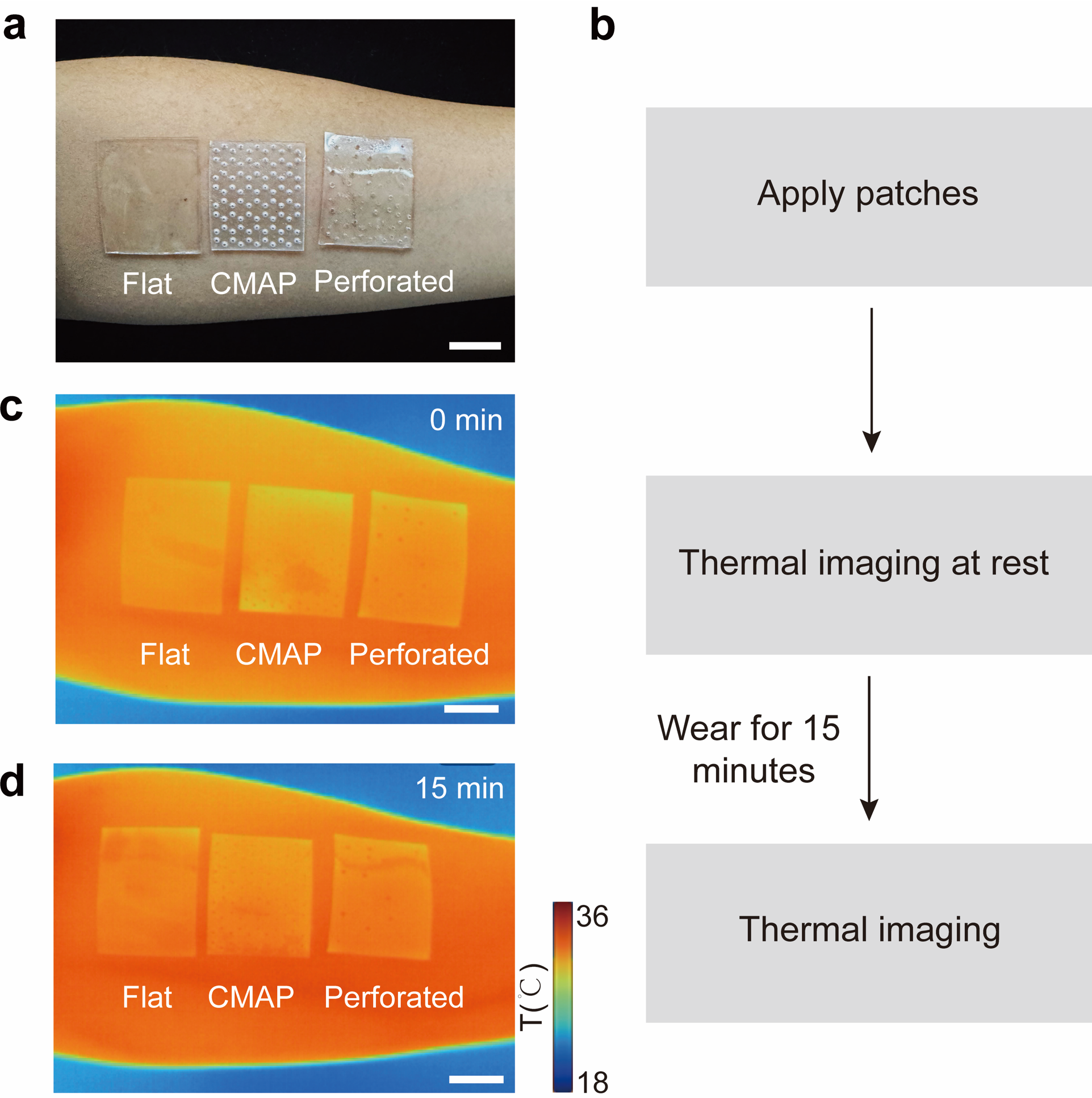


**Figure S19.** Thermal management comparison. **a** Optical image of the Flat (solid PDMS), CMAP (bioinspired), and Perforated (simple vertical pores) patches applied on the forearm. **b** Experimental protocol. **c-d** Infrared thermal images taken at rest (0 min) and after 15 minutes of wear. The CMAP patch exhibits the most effective cooling, confirming the contribution of active fluid transport beyond simple porosity.


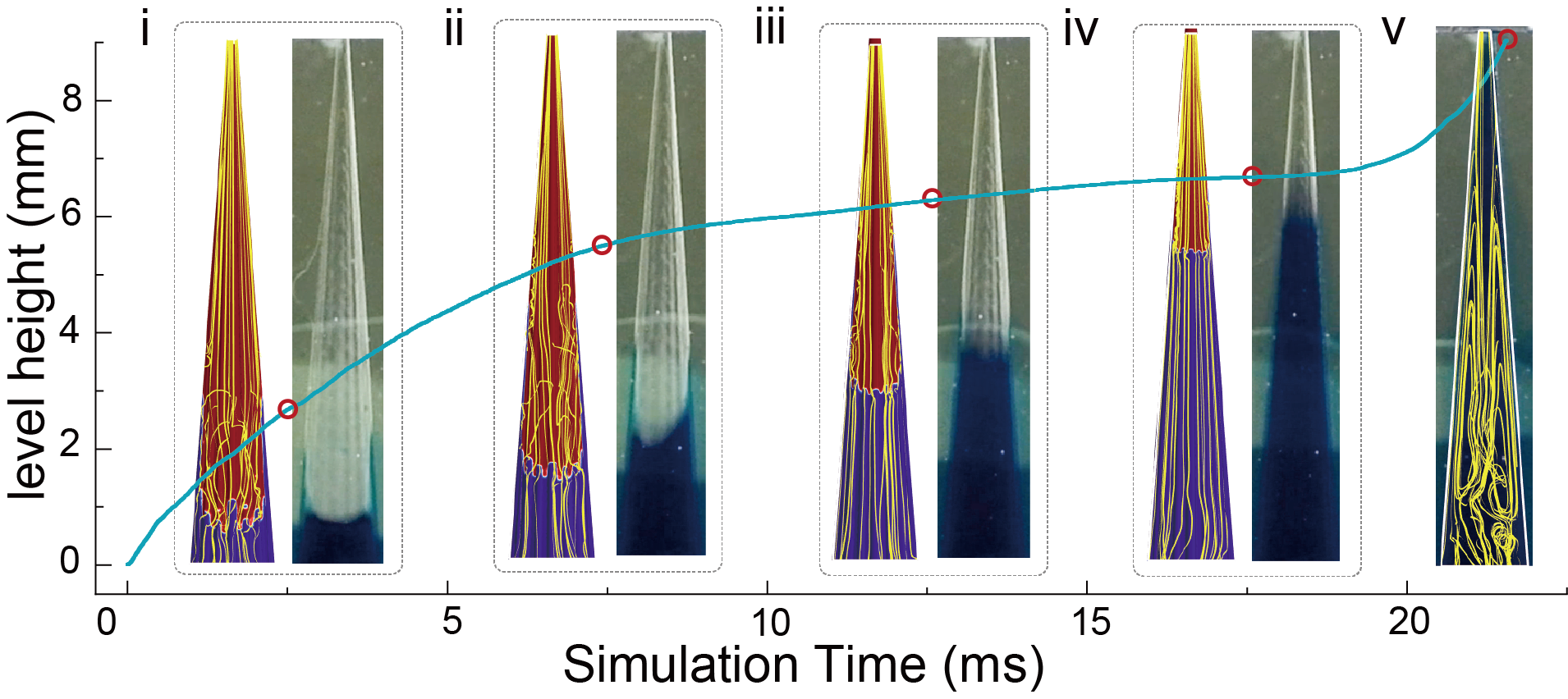


**Figure S20.** Comparison of vertical liquid rise between simulation and experiment, including interface height evolution and characteristic flow streamlines.


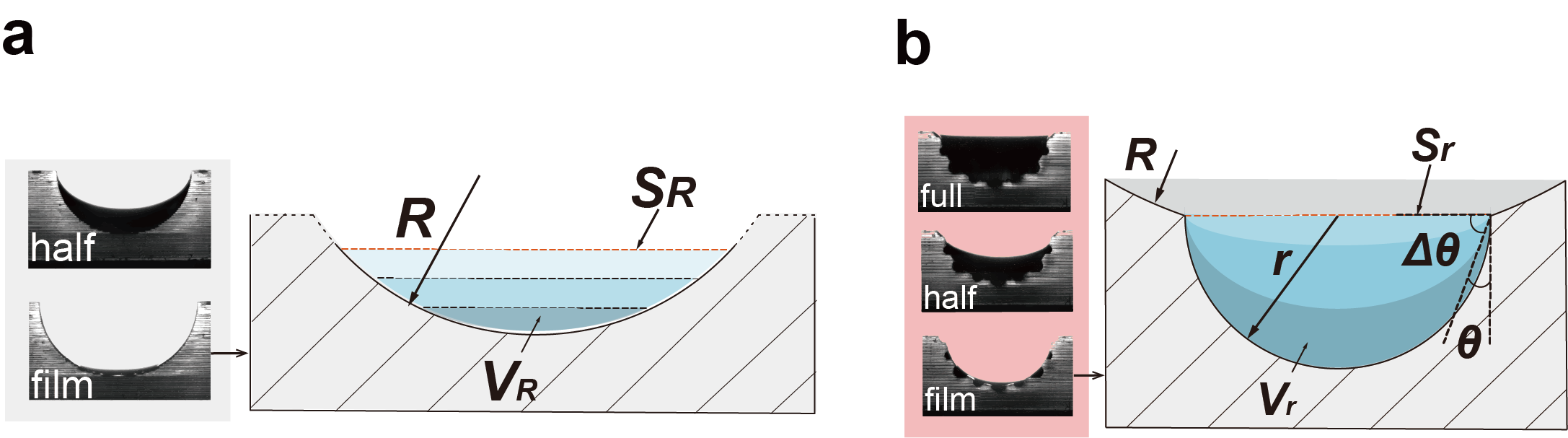


**Figure S21.** Photographs showing the evaporation evolution of liquid films on microgroove versus smooth structures. Different structures lead to two evaporation modes: constant contact angle evaporation and pinning evaporation.


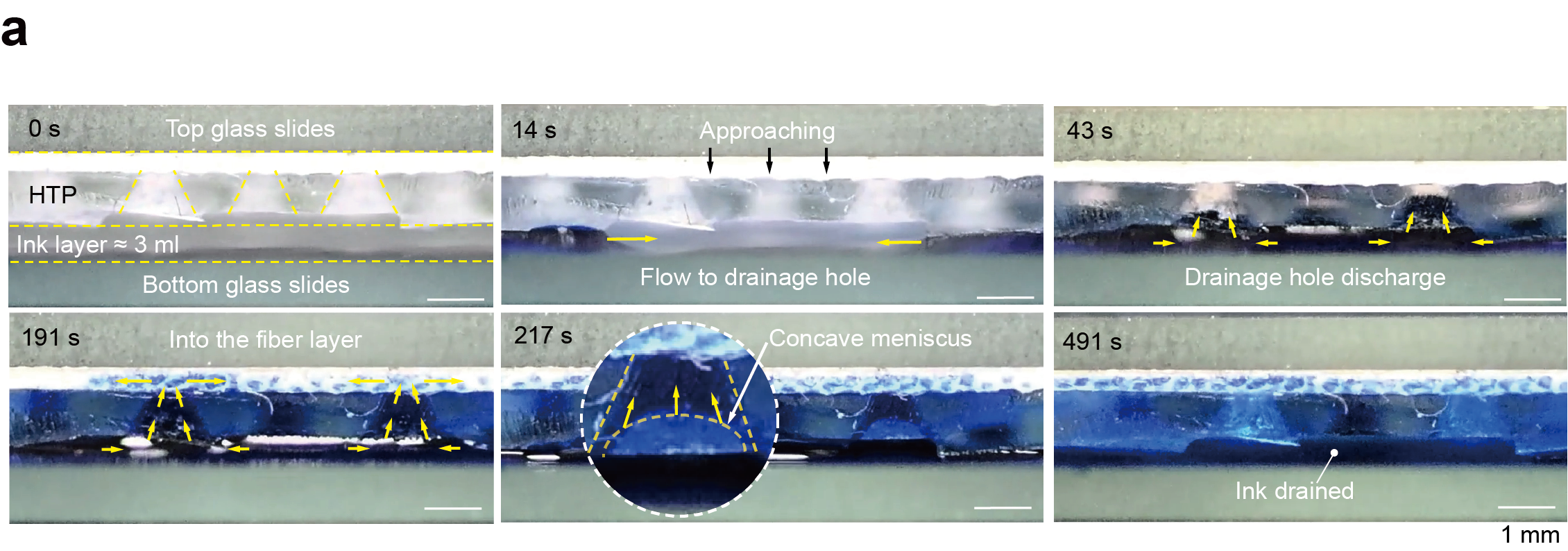


**Figure S22 .** Process of the liquid film transporting from the bottom through the CMAP to the top fabric layer.

**
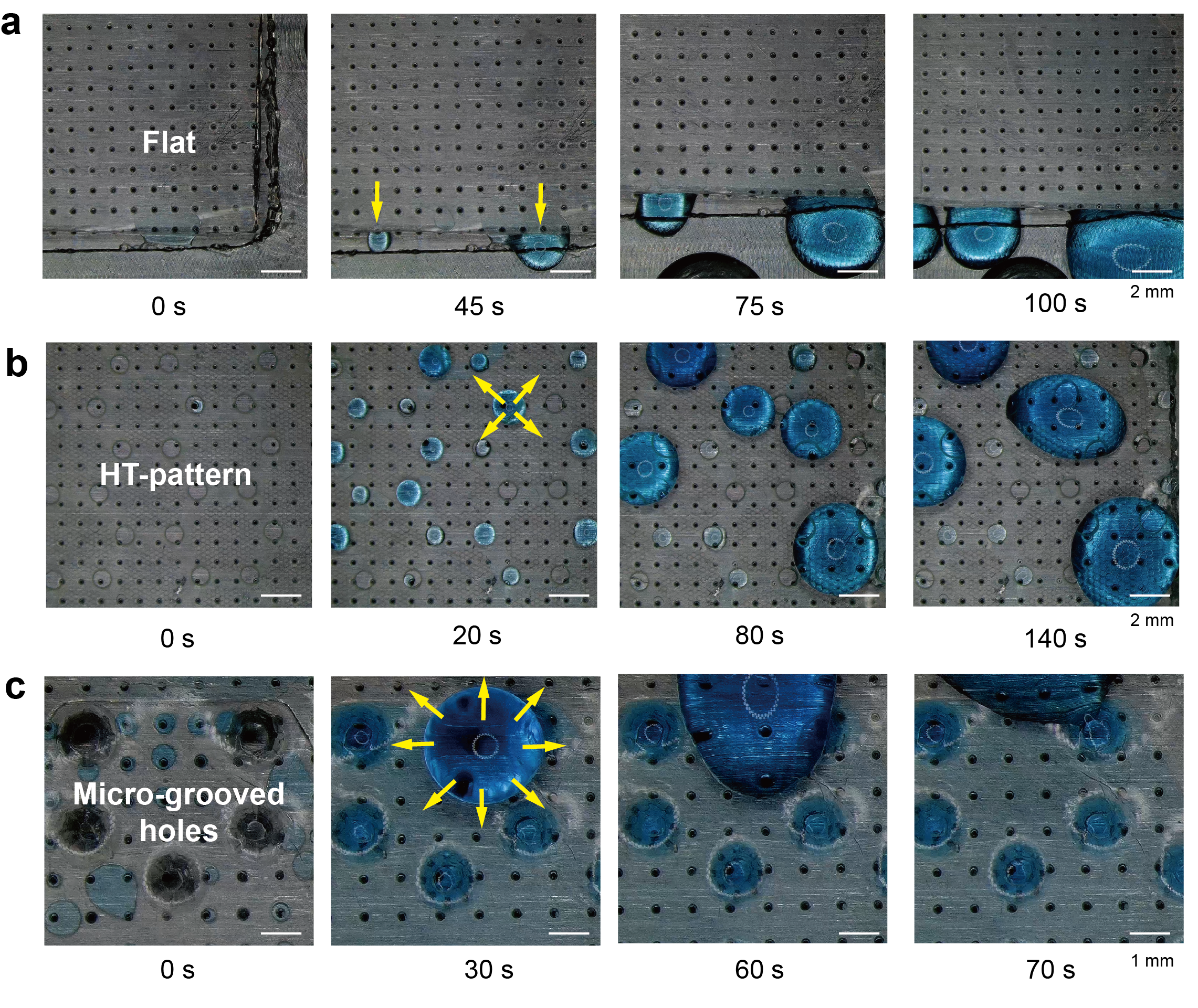
**

**Figure S23.** Sweat simulation process on flat patches, HT-perforated patches, and microgroove-equipped patches using a sweat simulation device.


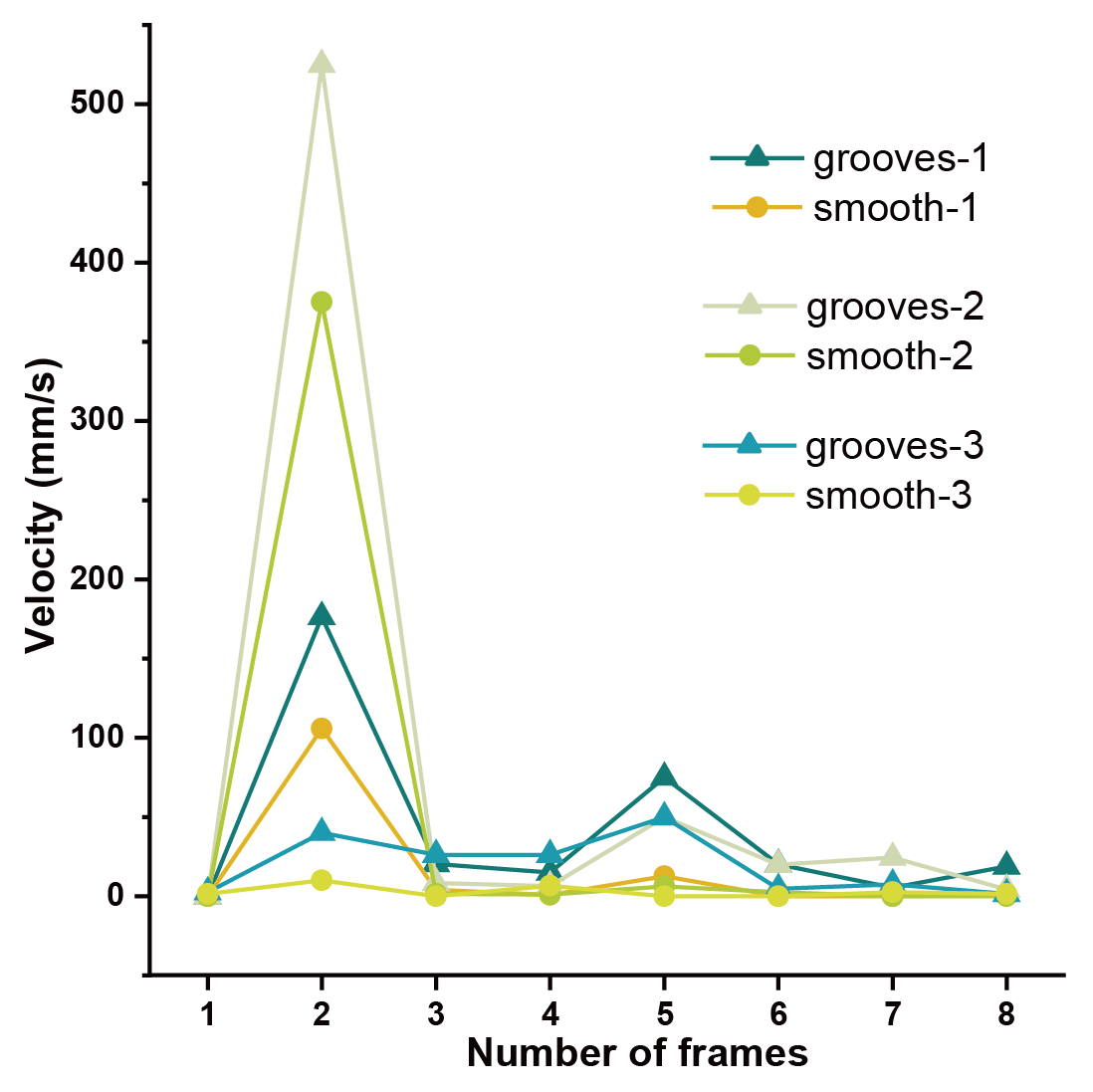


**Figure S24.** Velocity–frame curves of liquid propagation in asymmetric structures (double-level microgrooves vs. smooth surface).The microgroove structure accelerates liquid movement, contributing to a degree of unidirectional transport.


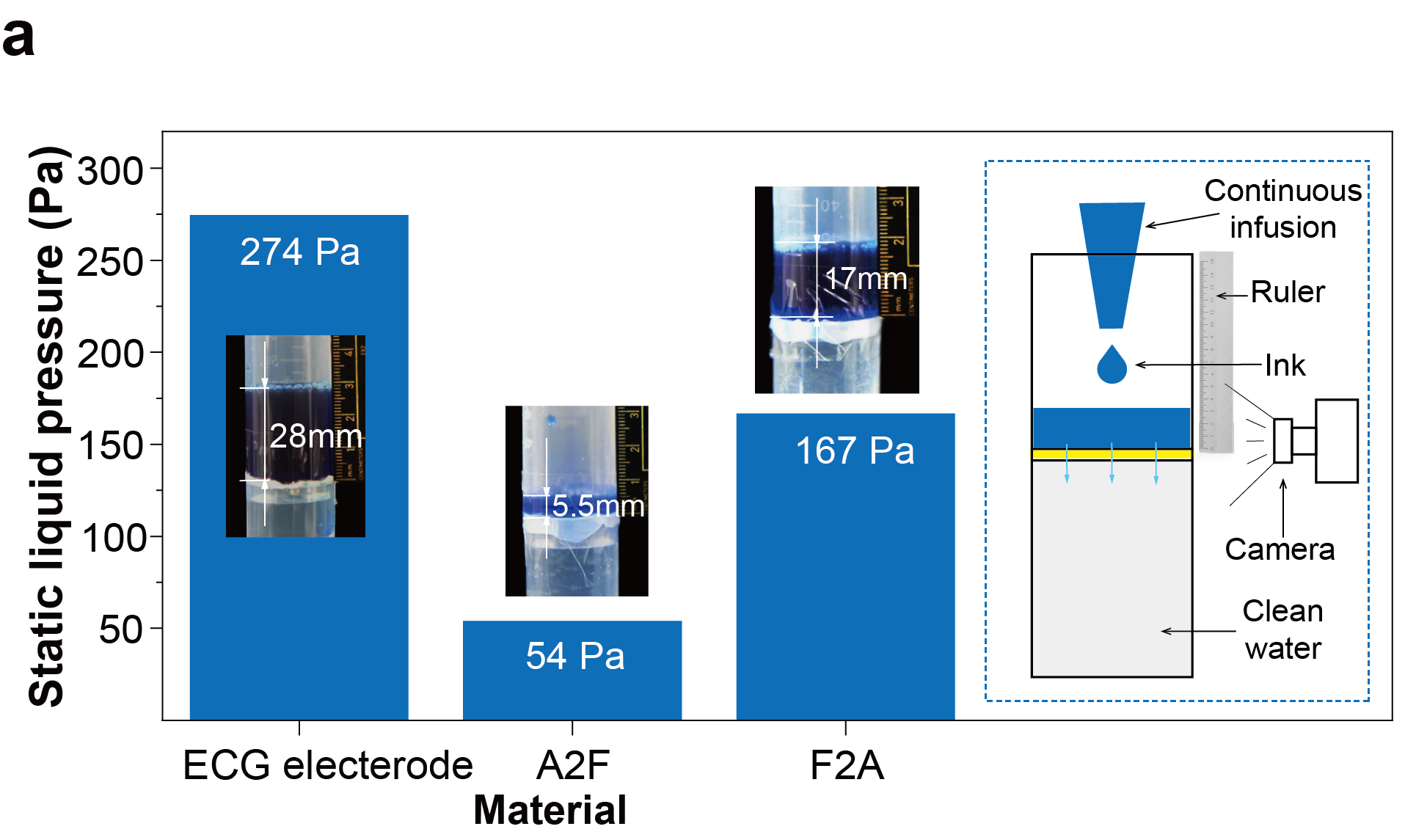


**Figure S25.** Comparison of pressure resistance tests between commercial ECG patches and CMAP membranes at both ends.

**
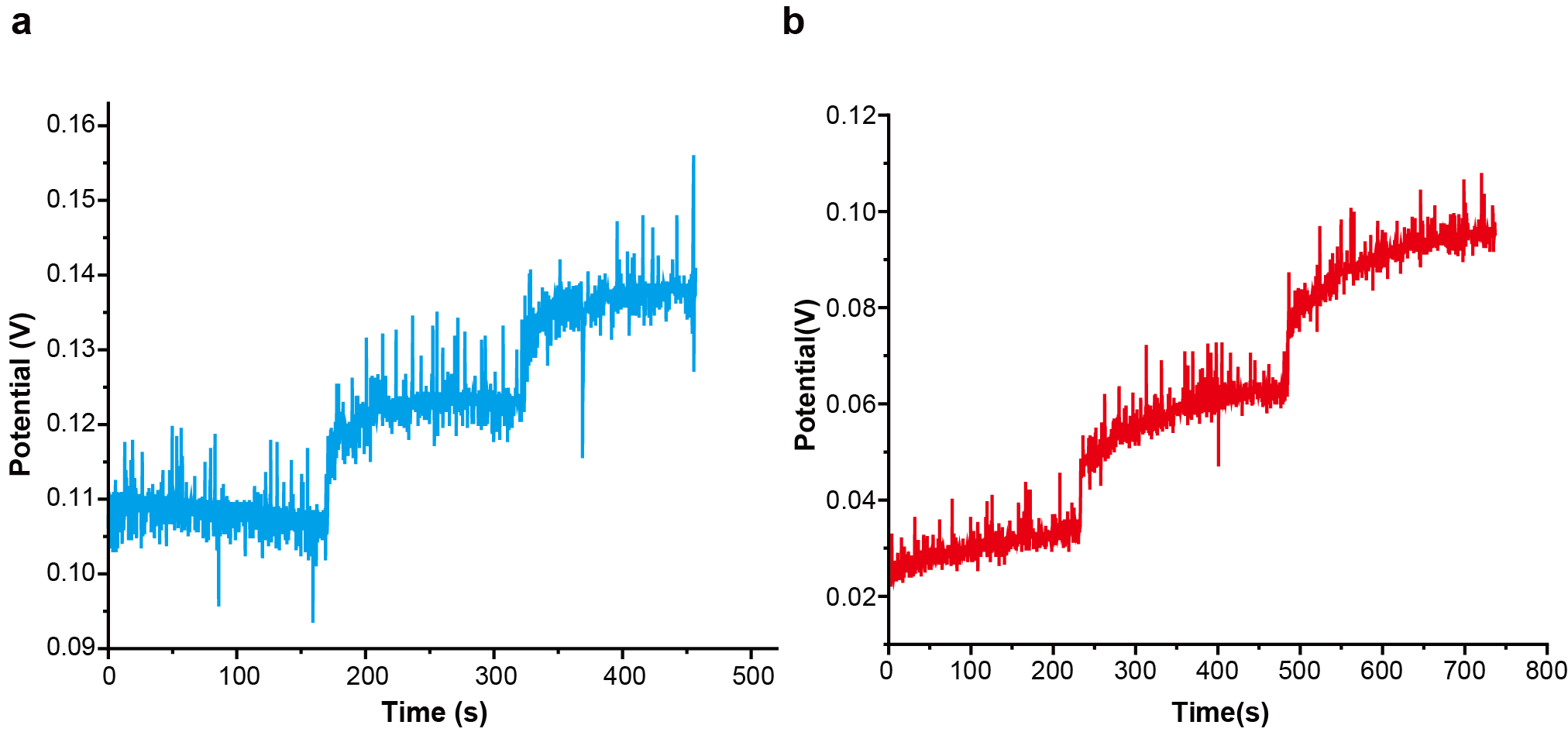
**

**Figure S26.** Voltage–concentration calibration of the sweat detection circuit using standard Ca²⁺ and Na⁺ solutions.**a** Ca²⁺ calibration performed with concentrations of 1, 2, and 4 mmol/L.**b** Na⁺ calibration performed with concentrations of 25, 50, and 100 mmol/L.


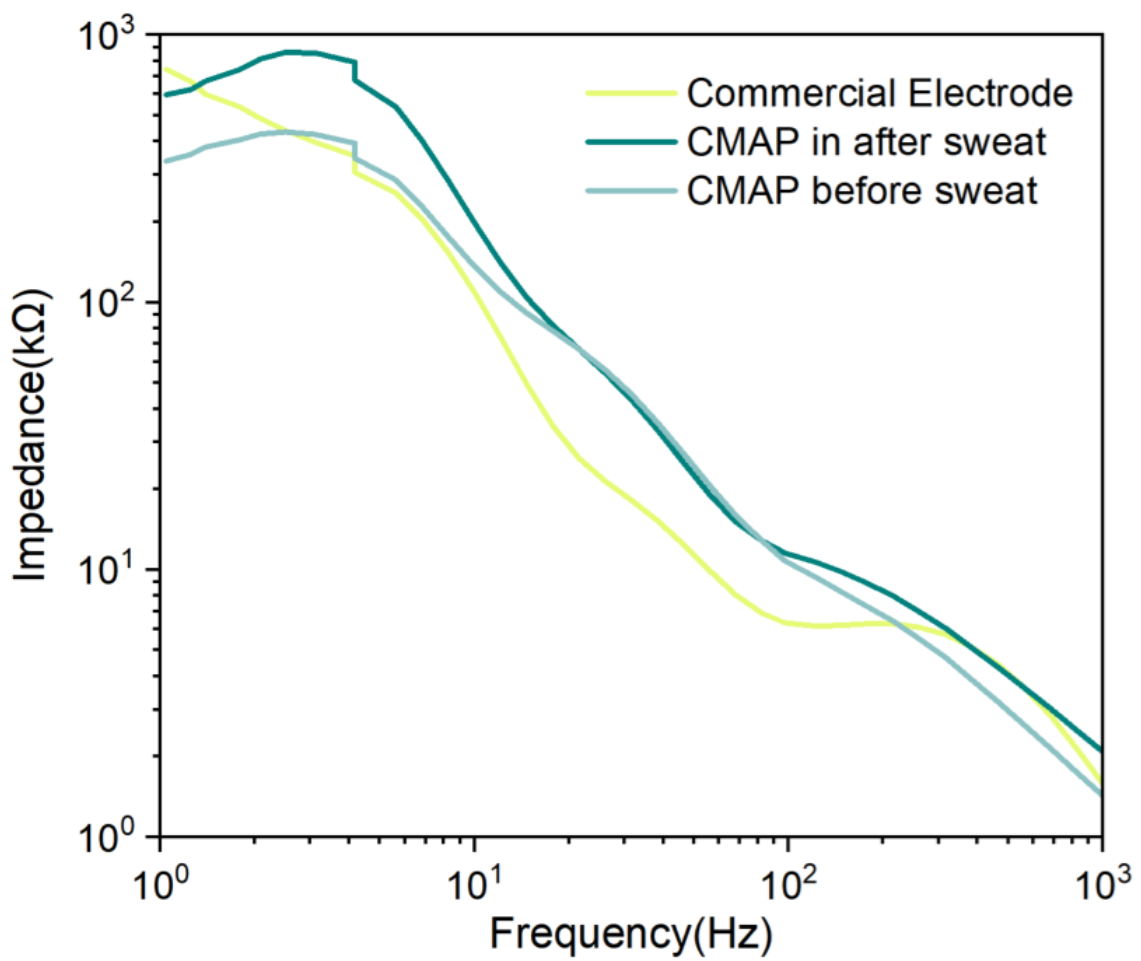


**Figure S27.** Comparison of skin-electrode interface impedance spectra (1 Hz–1 kHz) for a commercial Ag/AgCl electrode and the CMAP electrode under dry (before sweat) and wet (after sweat) conditions.


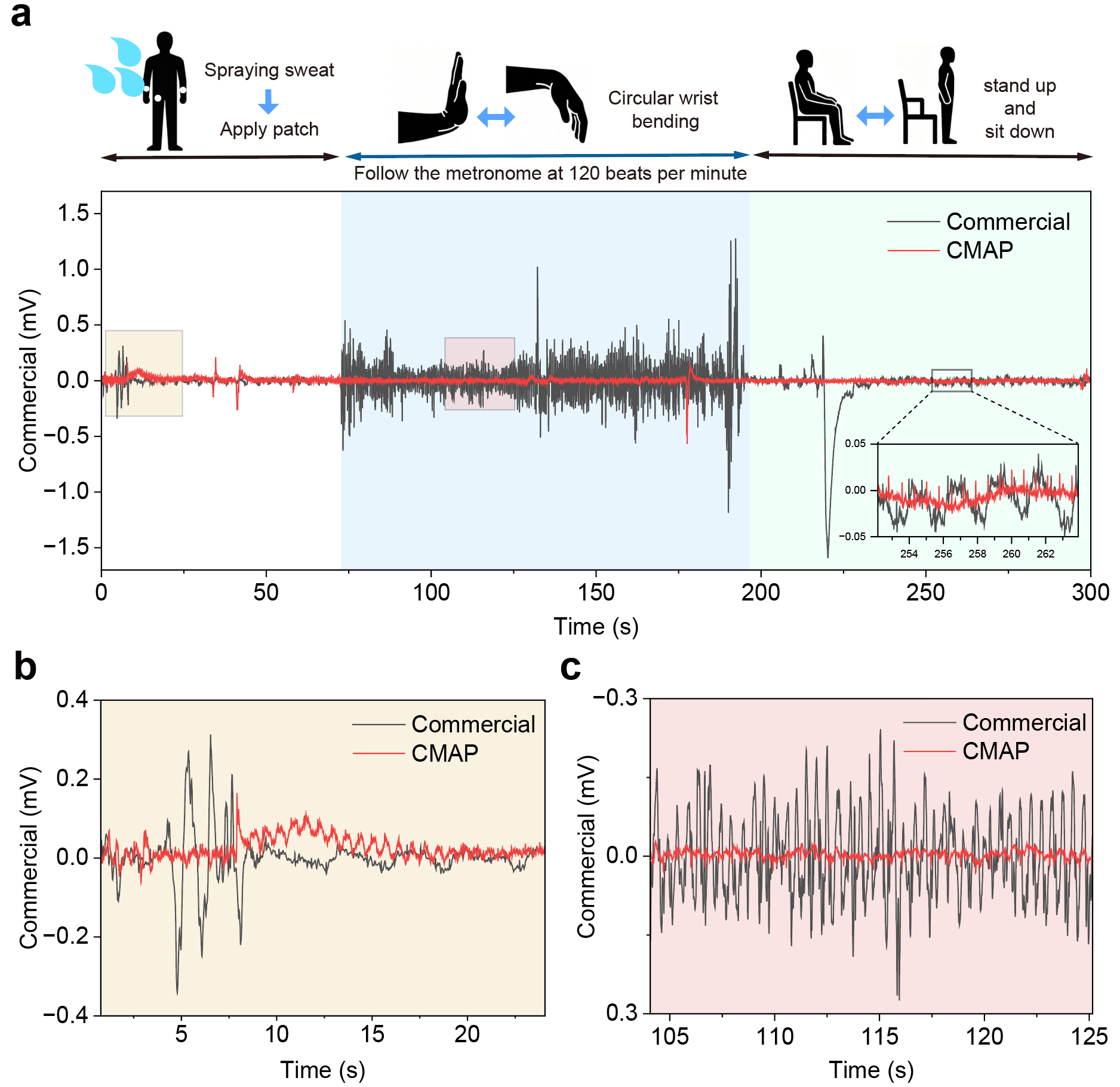


**Figure S28.** Comparison of ECG signal quality under dynamic conditions. **a** Experimental protocol and real-time recordings involving sweating, wrist flexion, and body movement. Red: CMAP; Black: Commercial electrode. **b, c** Magnified views showing significant baseline drift and motion artifacts in the commercial electrode during initial application on sweating skin (b) and wrist flexion (c), whereas the CMAP signal remained stable.


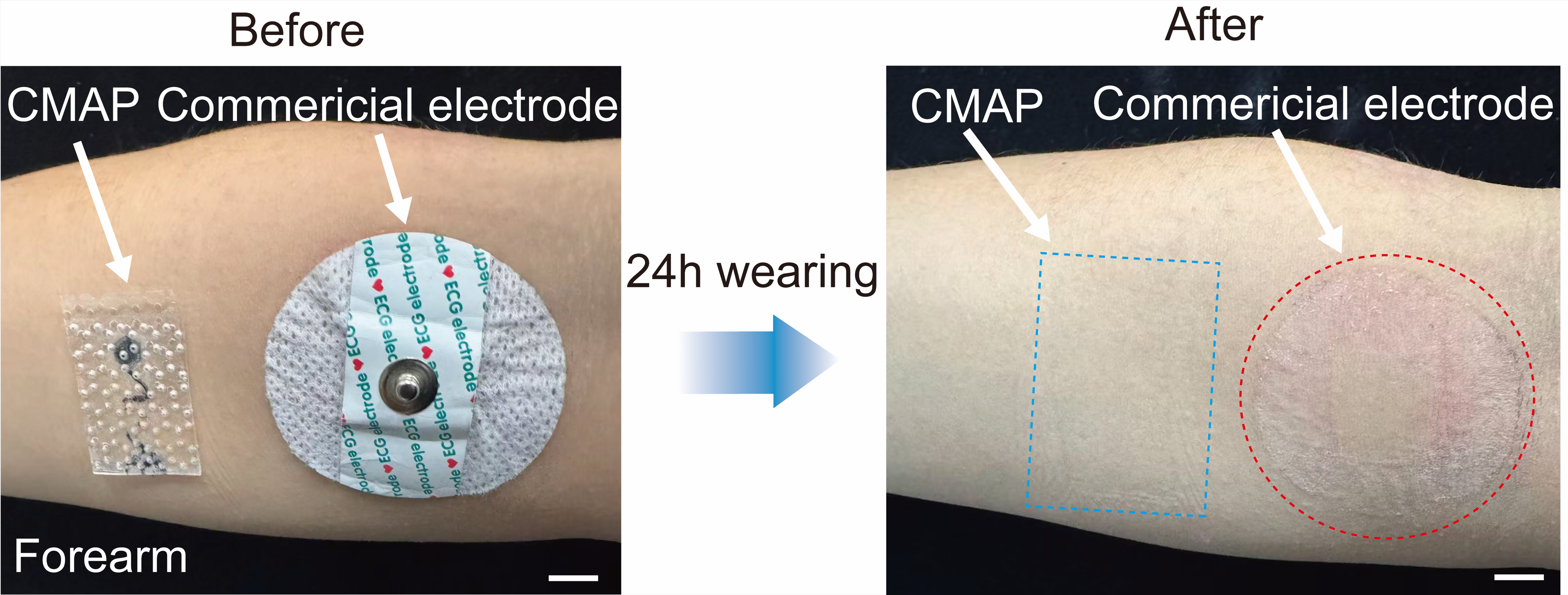


**Figure S29.** Skin status after 24-hour wear. The commercial electrode (red circle) caused maceration and erythema, whereas the CMAP area (blue square) remained healthy.


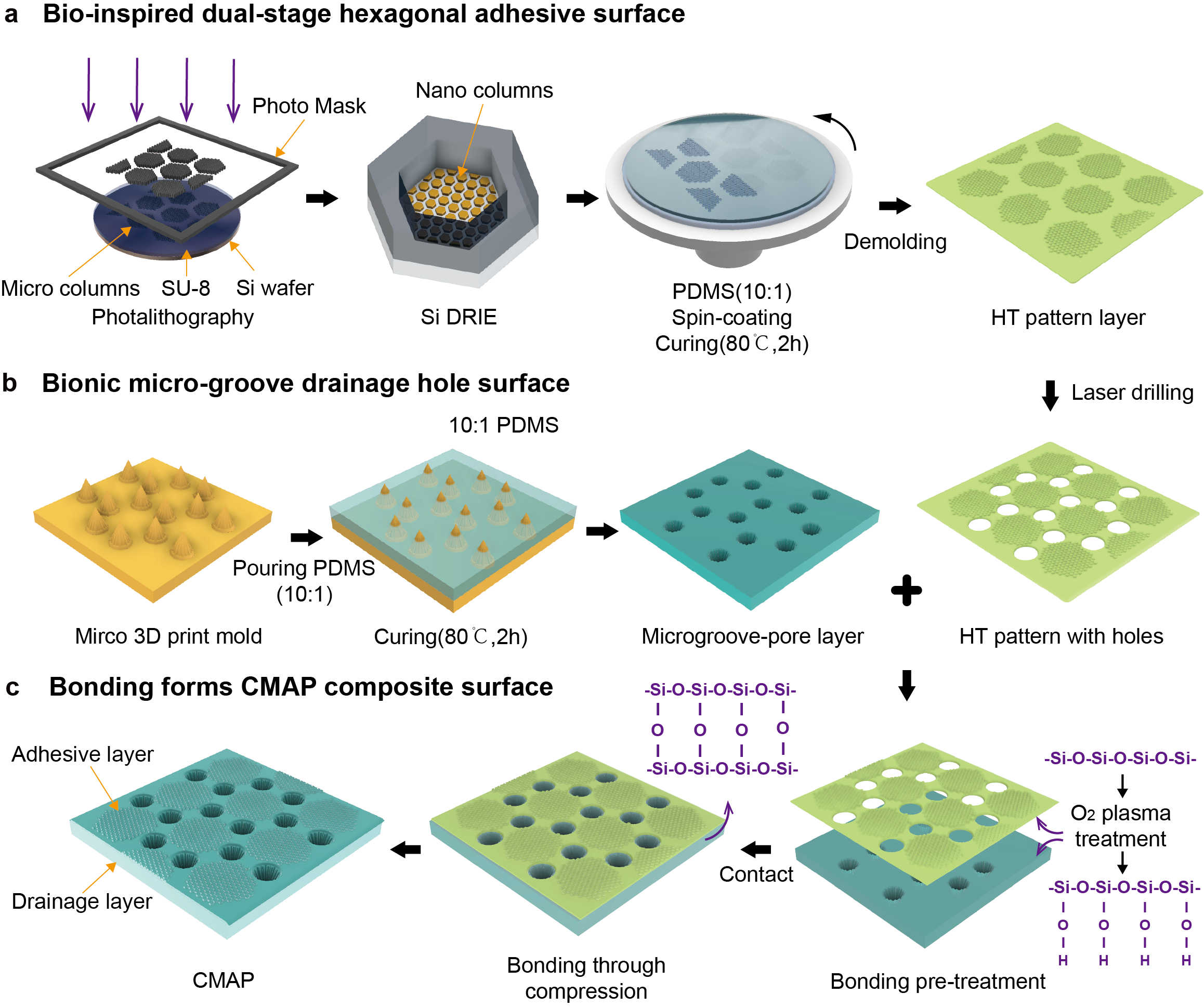


**Figure S30.** Fabrication process of the CMAP surface:**a** Photolithography and etching for preparing the second-order composite structure inspired by bees and tree frogs.**b** Fabrication of the microgroove transport channel layer. **c** Bonding to achieve integration of the dual-structure layers.


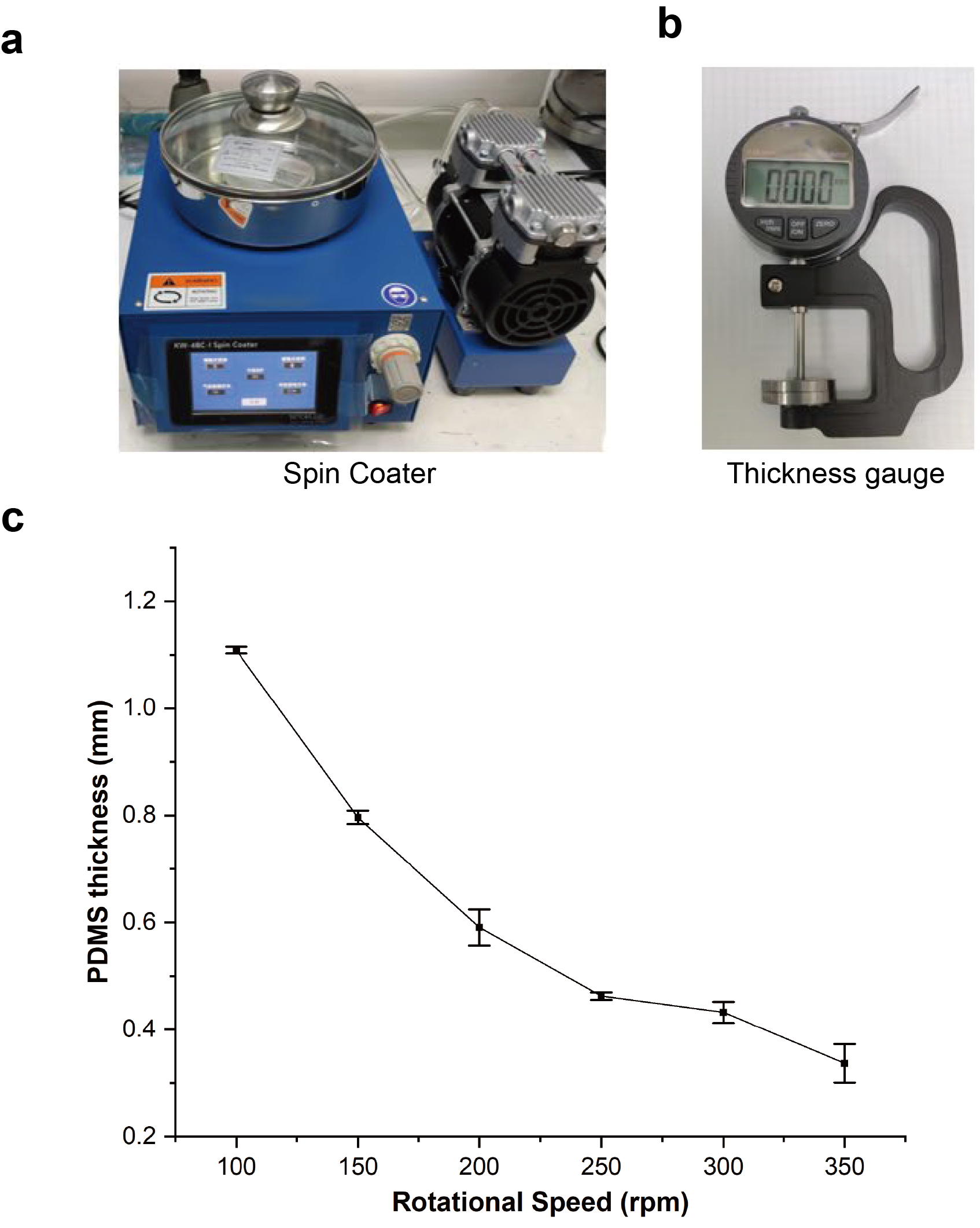


**Figure S31.** Photographs of micro/nano-printed molds used for fabricating conical structures.**a–c** show the mold structures of a smooth conical pore, a single-groove conical pore, and a double-groove conical pore, respectively.


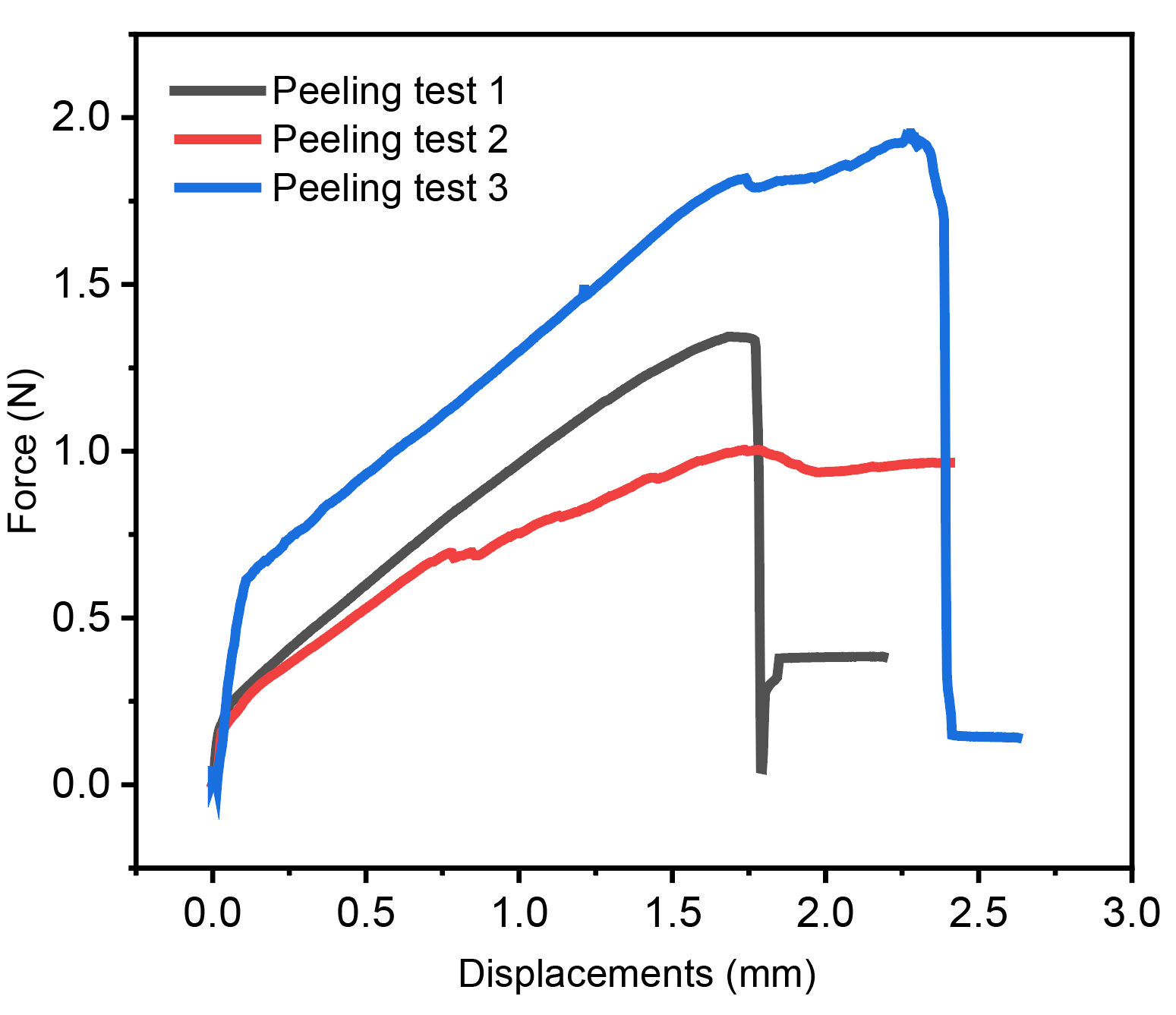


**Figure S32.** Destructive peel test results. The abrupt force drop without a stress plateau indicates cohesive failure within the PDMS, confirming that the interfacial bonding strength exceeds the material's tensile strength.


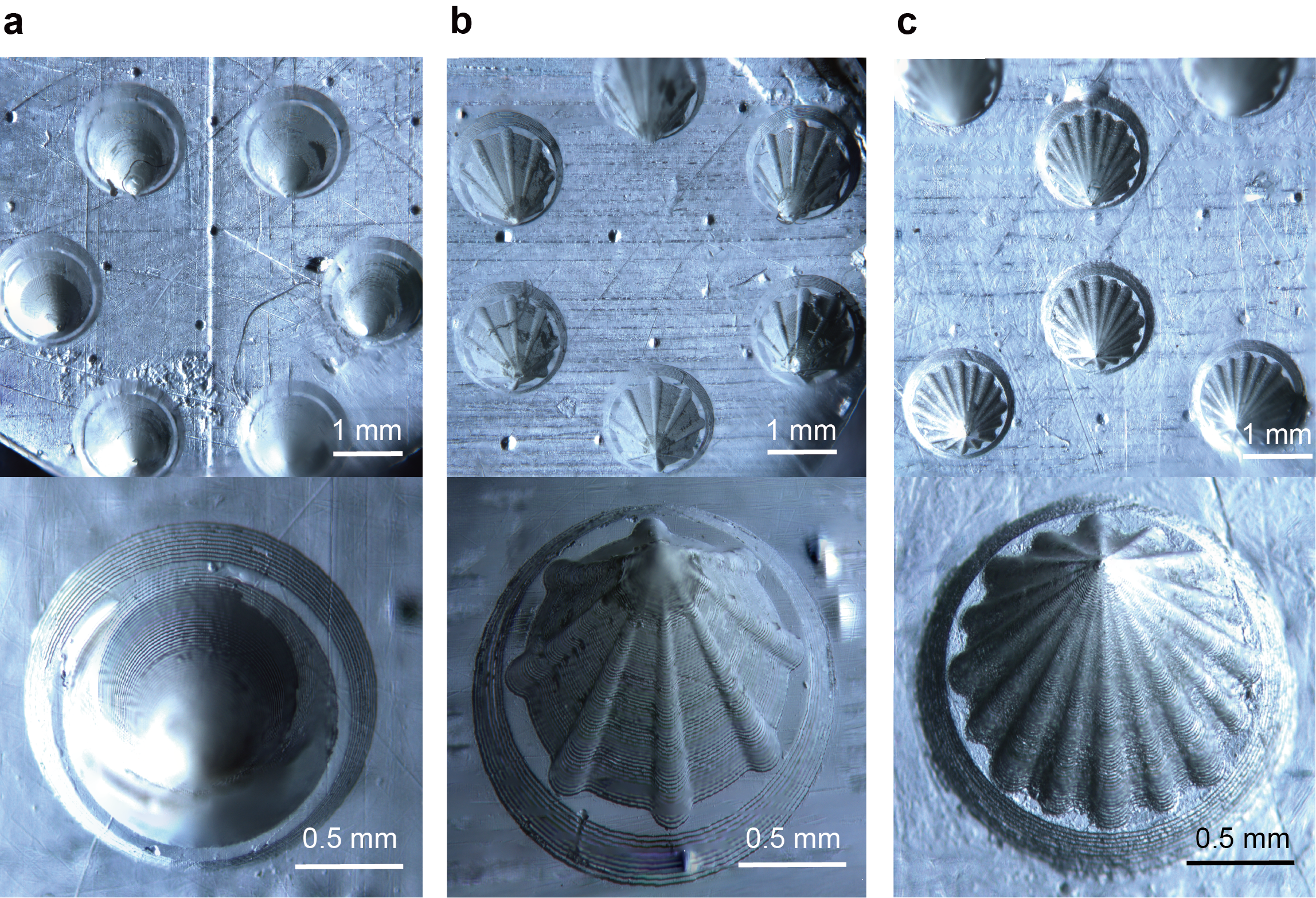


**Figure S33.** Photographs of micro/nano-printed molds for fabricating conical pore structures.**a–c** show the mold structures for a smooth conical pore, a single-groove conical pore, and a double-groove conical pore, respectively.


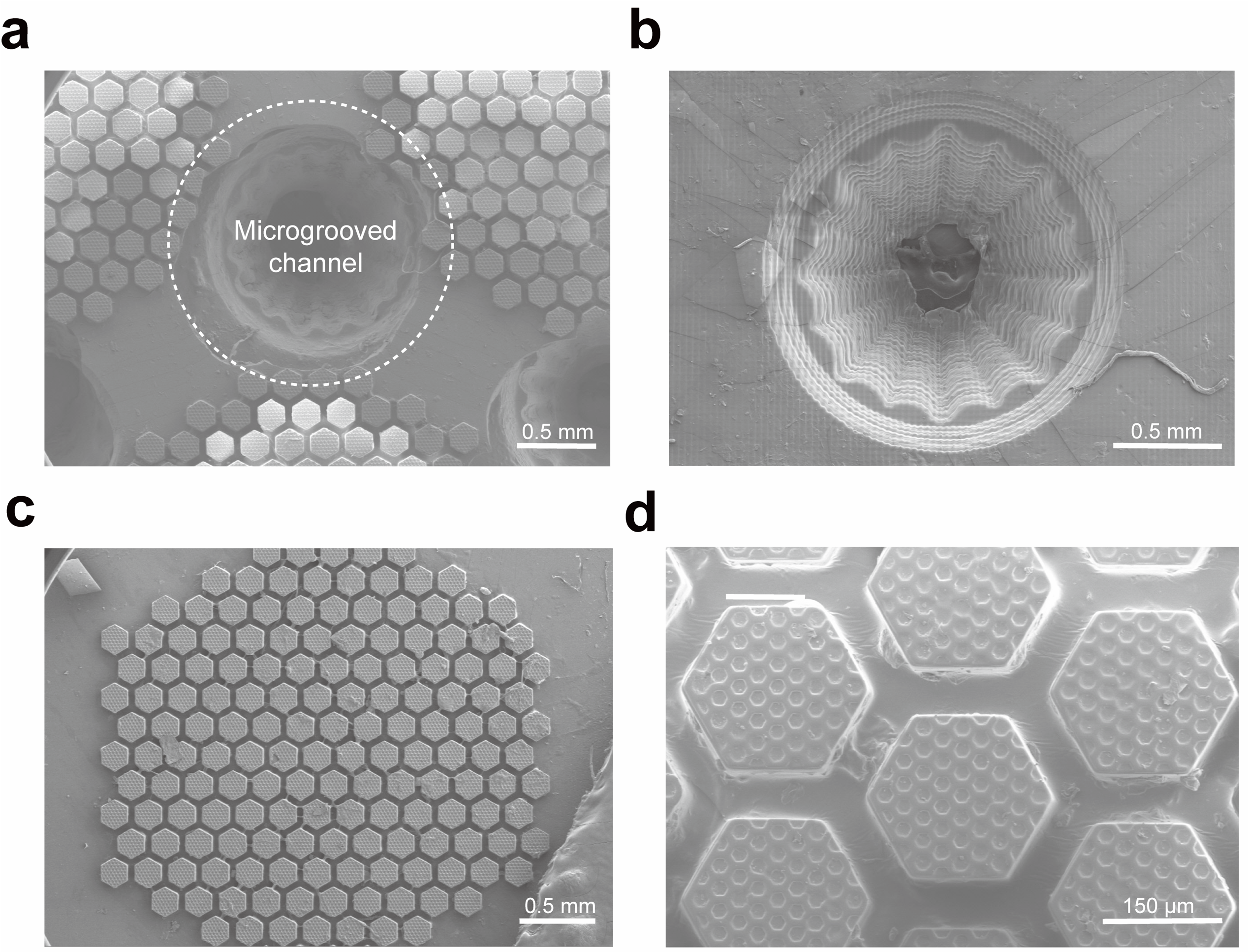


**Figure S34.** SEM characterization of the hierarchical CMAP architecture. **a** Overview of drainage pores and hexagonal pillars. **b** Close-up of microgrooved conical pores. **c** Tilted view of the pillar array. **d** High-magnification of surface micro-dimples.


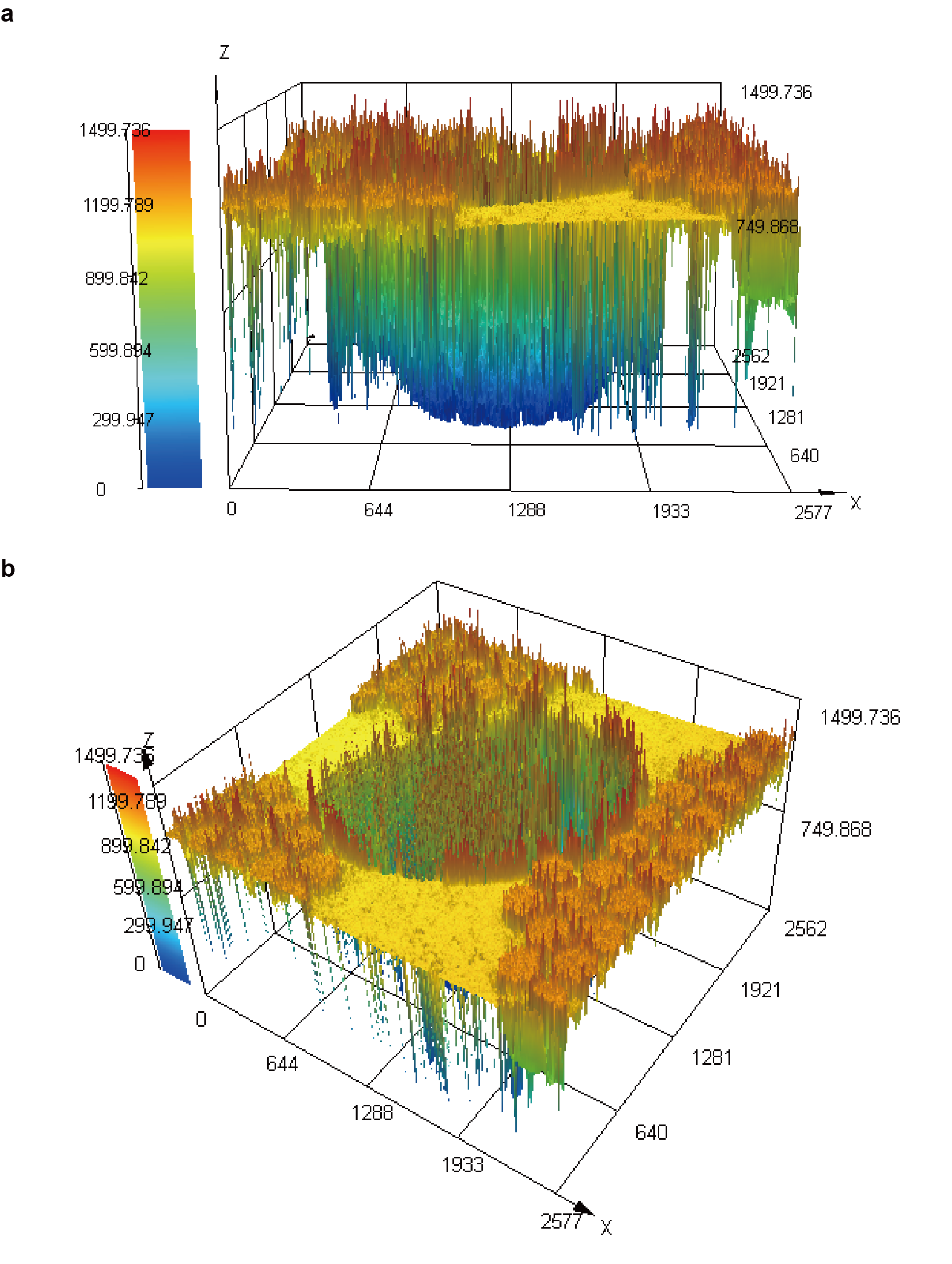


**Figure S35.** CLSM image of the CMAP patch drainage hole–hexagonal adhesion composite structure.

**Table S1. Boundary conditions of simulation**

| **Parameter** | **Symbol** | **Value** | **Description** |
| --- | --- | --- | --- |
| Density | $\rho$ | $997\text{ kg/m}^{3}$ | Standard density of sweat/water at 25 °C |
| Surface Tension | $\gamma$ | $0.072\text{ N/m}$ | Water-Air interface tension |
| Contact Angle (PVA-modified) | $\theta$ | 35° | Experimental value after O_2_ plasma + PVA treatment |
| Gravity | $g$ | $9.8\text{ m/s}^{2}$ | Gravitational acceleration |
| Atmospheric Pressure | $p_{atm}$ | $101.325\text{ kPa}$ | Reference ambient pressure for the outlet boundary |
| Navier Slip Factor | $f_{h}$ | 1 | Factor defining slip length proportional to mesh size |

**Table S2. Selectivity coefficients (Kij)**

| Log *K_ij_* | | Interference ion (*j*) | | |
| --- | --- | --- | --- | --- |
|  |  | NH_4_^+^ | Ca^2+^ | K^+^ |
| Target ion  (*i*) | Na^+^ | -3.216 | -2.382 | -3.441 |
|  |  | Mg^2+^ | NH_4_^+^ | Na^+^ |
|  | K^+^ | -2.026 | -2.080 | -2.234 |
|  |  | Mg^2+^ | K^+^ | Na^+^ |
|  | Ca^2+^ | -2.641 | -3.828 | -2.585 |

**Table S3. Influence of the PH (4–8)**

|  |  | PH=4 | PH=6 | PH=8 |
| --- | --- | --- | --- | --- |
| Na^+^ | Sensitivity | 62.47 | 60.02 | 58.62 |
|  | R^2^ | 0.995 | 0.996 | 0.994 |
| Ca^2+^ | Sensitivity | 40.44 | 48.78 | 42.89 |
|  | R^2^ | 0.999 | 0.998 | 0.997 |
| K^+^ | Sensitivity | 76.71 | 68.12 | 61.67 |
|  | R^2^ | 0.995 | 0.990 | 0.985 |

**Table S4. Performance comparison of CMAP and representative epidermal interfaces.**

|  | **CMAP(This work)** | **Web Electrode**[6] | **MN-Patch**[7] | **Janus-T**[8] | **Nanomesh**[9] | **Commercial** |
| --- | --- | --- | --- | --- | --- | --- |
| Type / Key Feature | Bioinspired Hybrid  Tree Frog + Pitcher Plant structure with active pumping. | Flexible electrode with dispersed hexagonal pillars and asymmetric cone holes | PDMS-t adhesive with conical through-holes, hexagonal microgrooves, and Ag/Ni microneedles | Hydrophobic (polyester) / superhydrophilic (nitrocellulose) textile with conical micropores. | Gold-coated polyvinyl alcohol (PVA) nanofibers that form a mesh directly on the skin. | Standard Hydrogel  (Solid Elastomer) |
| **Wet Adhesion Stability** | ★★★★☆ | ★★★☆☆ | ★★★★★ | ★★★☆☆ | ★★★☆☆ | ★★★☆☆ |
|  | Mech Air-plug effect、Capillarity  ~2.5 kPa @ 1N | Mech: Capillarity with hexagonal pillars  ~2.3 kPa @ 1N | Mech: Invasive Interlocking  ~12.5 kPa @ 1N | Mech: Surface Friction  Slippery in wet | Mech: Van der Waals  Easy peeling | Mech: Hydroplaning |
| **Sweat Transport Capability** | ★★★★★ | ★★★★☆ | ★★★★☆ | ★★★★☆ | ★★★★★ | ★★★☆☆ |
|  | Active Pumping with 2 directions  ~500 mm/s | Unidirectional through-holes: Pumps sweat out but lacks lateral spread pump. | Porous Capillarity: Good wicking but prone to saturation. | Textile Wicking: Unidirectional wicking for comfort; no pumping. | Gas Permeability: Passive evaporation; no directional liquid pumping. | None |
| **Multimodal Scalability** | ★★★★★ | ★★★☆☆ | ★★★☆☆ | ★N/A | ★★★☆☆ | ★★★☆☆ |
|  | High: ECG, EMG, GSR, and Sweat Ion sensing. | Moderate: Mainly Electrophysiology (ECG/EMG/EEG). | Moderate: Physiological signals (ECG). | Focus on fluid management | Moderate: Mainly EMG and touch/temp sensors. | ECG only |
| **Biocompatibility** | ★★★★★ | ★★★★☆ | ★★★☆☆ | ★★★★★ | ★★★★★ | ★★★☆☆ |
|  | Non-invasive: High safety profile. | Potential Cytotoxicity: Uses imidazole-based ionic liquids which may irritate. | Potential Irritation: Invasive nature of microneedles may cause discomfort. | Textile comfort: Designed for thermal management and wearability. | Inflammation-free: Excellent gas permeability minimizes rash risk. | Skin Maceration  Due to sweat accumulation |
| **Interfacial Impedance Stability** | ★★★★☆ | ★★★★★ | ★★★★★ | ★N/A | ★★★☆☆ | ★★★☆☆ |
|  | Stable in Flowing Water  ~45.3 kΩ @ 30Hz  Robust signal fidelity | Low impedance due to ionic liquids.  ~30 kΩ @ 10Hz | Lower than gel patches due to stratum corneum penetration.  ~100 kΩ@ 10Hz | Not designed as electrode | Higher impedance; signal drifts in heavy sweat.  ~140 kΩ @ 100Hz | ~120 kΩ (Dry)  Signal loss / Artifacts) |

**Table S5. Definition of Scoring Criteria for Performance Comparison**

| **Dimension** | **★★★★★**  **(Excellent)** | **★★★☆☆**  **(Moderate)** | **★☆☆☆☆**  **(Poor)** |
| --- | --- | --- | --- |
| **Wet Adhesion Stability** | Ultra-Robust / Invasive Shear strength > 10 kPa | Shear strength ~0.2–10 kPa | Failure Immediate detachment or hydroplaning upon wetting |
| **Sweat Transport** | Active Pumping Directional velocity > 100 mm/s driven by Laplace pressure or gradient | Passive Wicking Capillary wicking via porous structures; no active driving force | Accumulation Zero transport; sweat traps at the interface |
| **Multimodality** | Scalable Multimodal Capability System is capable of integrating simultaneous electrophysiological and biochemical sensing modules | Single Domain Restricted to either electrophysiology or simple fluid sampling. | No sensing capability. |
| **Biocompatibility** | Inflammation-Free Non-invasive, breathable, and prevents maceration via active drainage | Risk Factors Potential cytotoxicity (chemical) or physical trauma (invasive) | Skin Damage High risk of maceration, pruritus, or allergic reactions |
| **Impedance Stability** | Ultra-Low / Penetrating Impedance < 40 kΩ; stable under extreme wet/flowing conditions | Variable Impedance < 150 kΩ in dry; significantly drifts or degrades in wet states | Unstable Signal loss or severe artifacts under motion/wetting |

**References:**

1. Siqveland LM, Skjaeveland SM. Derivations of the Young-Laplace equation. Capillarity. 2021;4:23–30. https://doi.org/10.46690/capi.2021.02.01

2. Meseguer J, Slobozhanin LA, Perales JM. A review on the stability of liquid bridges. Advances in Space Research. 1995;16:5–14. https://doi.org/10.1016/0273-1177(95)00126-Y

3. Van Nguyen P, Luu QK, Takamura Y, Ho VA. Wet Adhesion of Micro-patterned Interfaces for Stable Grasping of Deformable Objects. 2020 IEEE/RSJ International Conference on Intelligent Robots and Systems (IROS) [Internet]. Las Vegas, NV, USA: IEEE; 2020 [cited 2025 May 29]. p. 9213–9. https://doi.org/10.1109/IROS45743.2020.9341095

4. Ma Z, Liang L, Zhang C, Xiang Y, Yan M, Liu Z, et al. Bionic Wet Adhesive Surface with Micronano Hierarchical Structure for Robust Biosignal Monitoring. ACS Appl Mater Interfaces. 2025;17:20471–82. https://doi.org/10.1021/acsami.5c02890

5. COMSOL Multiphysics. Capillary Filling — Level Set Method [Internet]. Stockholm,SE; 2024. https://cn.comsol.com/model/capillary-filling-1878

6. Lan T, Tian H, Chen X, Li X, Wang C, Wang D, et al. Treefrog‐Inspired Flexible Electrode with High Permeability, Stable Adhesion, and Robust Durability. Advanced Materials. 2024;2404761. https://doi.org/10.1002/adma.202404761

7. Zhang Q, Ji K, Huo T, Khan MN, Hu Z, Yuan C, et al. Biomimetic Patch with Wicking-Breathable and Multi-mechanism Adhesion for Bioelectrical Signal Monitoring. ACS Appl Mater Interfaces. American Chemical Society; 2022;14:48438–48. https://doi.org/10.1021/acsami.2c13984

8. Dai B, Li K, Shi L, Wan X, Liu X, Zhang F, et al. Bioinspired Janus Textile with Conical Micropores for Human Body Moisture and Thermal Management. Advanced Materials. 2019;31:1904113. https://doi.org/10.1002/adma.201904113

9. Miyamoto A, Lee S, Cooray NF, Lee S, Mori M, Matsuhisa N, et al. Inflammation-free, gas-permeable, lightweight, stretchable on-skin electronics with nanomeshes. Nature Nanotech. 2017;12:907–13. https://doi.org/10.1038/nnano.2017.125
